# Catalyst-Free Bulk Depolymerization of Polymethacrylates Prepared by ATRP

**DOI:** 10.1021/acsmacrolett.6c00380

**Published:** 2026-09-10

**Authors:** Brendan Pozzi, Stella Afroditi Mountaki, Richard Whitfield, Athina Anastasaki

**Affiliations:** Laboratory of Sustainable Polymers, Department of Materials, ETH Zurich, Vladimir-Prelog-Weg 5, 8093 Zurich, Switzerland

## Abstract

Low-temperature depolymerization of ATRP polymers has been successfully demonstrated both in bulk and solution. However, all reported examples necessitate a metal catalyst for chain-end activation, require extensive modifications and transformations prior to depolymerization, or use specialized initiators. Herein, a catalyst-free bulk depolymerization method for poly­(*O*-aryl methacrylates) is realized, whereby the elimination of lactonization lowers the temperature by more than 100 °C and enables up to 92% depolymerization efficiency at temperatures as low as 230 °C. Key to the approach is the in-situ thermal transformation of halogen chain-ends into macromonomer, resulting in monomer yields comparable to those obtained in the presence of a catalyst. The method can be expanded from various poly­(*O*-aryl methacrylates) to conventional polymethacrylates, for example, poly­(methyl methacrylate) and poly­(benzyl methacrylate), after a short block addition of an *O*-aryl methacrylate. This work provides a straightforward, high-yielding, catalyst-free bulk method for chemical recycling and offers mechanistic insights into the thermal stability and depolymerization of ATRP polymers.

## Graphical abstract

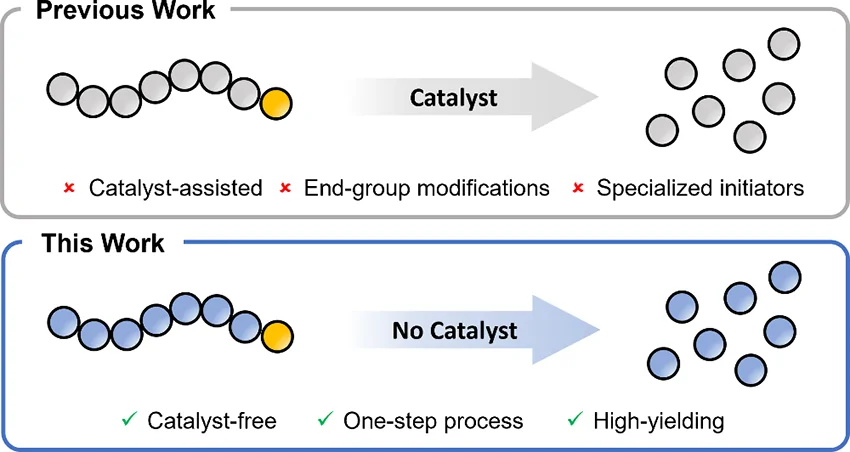

Since its conception in 1995, atom transfer radical polymerization (ATRP) has developed into an efficient tool for the preparation of precision polymers.^1,2^ The key step is the transfer of a halogen atom between a propagating radical and a transition-metal catalyst species, which facilitates uniform growth of the polymer chains and generates materials with high end-group fidelity.^3–5^ This high livingness has been widely exploited to synthesize bespoke materials with complex architectures, for example, multiblock copolymers, while also allowing quantitative end-group functionalization.^6–12^ Most recently, under thermodynamically favorable conditions, it has been demonstrated that these halogen end groups can be activated to achieve low-temperature depolymerization.^13–19^

The first hint that depolymerization of ATRP materials was feasible was reported by Raus and coworkers, who identified a plateau in the polymerization of a polyhedral oligomeric silsesquioxane methacrylate after the reaction temperature was increased from 60 to 90 °C.^20^ The Ouchi group achieved the first thermal depolymerization of poly­(methyl methacrylate) (PMMA), retrieving up to 24% of the starting monomer by utilizing a chlorine terminal polymer in the presence of a ruthenium catalyst, initiating extensive research in the area.^21^ Matyjaszewski and co-workers achieved much higher monomer yields during the depolymerization of polymers containing either bulky or non-bulky monomers (60–80%).^22–24^ The key to these approaches was the use of either copper or iron catalysts at 170 °C alongside chlorine terminal polymers, which allowed rapid chain-end activation and efficient depropagation.^25^ Our group subsequently illustrated that bromine-terminated polymers could also be successfully depolymerized in the presence of an iron catalyst and efficient depolymerization has also been reported under oxygen-tolerant conditions.^26–29^ Alternative methods have been developed that lower the temperature of depolymerization to 120 °C by utilizing either a copper or iron catalyst in combination with either a small amount of radical initiator, electrochemistry or the presence of blue light.^30–33^ This has allowed near-quantitative monomer regeneration and, in the latter cases, the opportunity to introduce temporal control into depolymerization reactions.^30^ Altogether, these are excellent developments, and high-yielding, rapid depolymerization of ATRP polymers is now commonplace. It is noted that the overwhelming majority of these reports (and also reports for the depolymerization of polymers produced by Reversible Addition–Fragmentation Chain Transfer Polymerization (RAFT))^34,35^ take place in solution and not only require the use of a solvent, but also can exclusively operate under diluted concentrations, thus, potentially limiting the depolymerization scope. To overcome this, bulk depolymerization approaches have been alternatively developed for polymers synthesized by ATRP. These methods not only avoid the use of a solvent but also allow high-purity monomer to be directly acquired typically through distillation.^36^ For example, our laboratory reported the modification of the halogen chain-end to macromonomer prior to depolymerization, allowing for subsequent low-temperature chemical recycling at 220 °C.^37^ Sumerlin and coworkers introduced a *N*-hydroxyphthalimide ester at the α-chain end, which could be thermally activated at 220 °C, achieving efficient depolymerization.^38,39^ In addition, the Matyjaszewski group demonstrated that the addition of α-terminal pyromellitic imide esters and phthalimide functionalities, alongside the catalytic activation of the ω-terminal halide groups, could significantly improve the depolymerization efficiency.^40^ It is noted, that the aforementioned reports either rely on end-group modifications, or the use of complex and specialized ATRP initiators. Instead, direct activation of the chain-end has to date only been reported by Matyjaszewski and co-workers, who elegantly demonstrated that a CuCl_2_/TPMA (tris­(2-pyridylmethyl)­amine) catalyst could directly activate the halogen at 230 °C, enabling rapid depolymerization and high monomer recovery up to 70%.^41^ Nevertheless, this inspiring report requires a metal complex (i.e., a metal salt and a ligand) for successful depolymerization to be achieved, potentially adding not only cost and toxicity, but also demanding careful optimization of the conditions.

Herein, we report a catalyst-free bulk depolymerization approach that provides high monomer yields at low temperatures. Elimination of a deleterious side reaction, namely, lactonization, is achieved, and the methodology is compatible with various poly­(*O*-aryl methacrylates), including poly­(phenyl methacrylate), poly­(cresol methacrylate), and poly­(guaiacol methacrylate). Notably, by addition of a short block of an *O*-aryl methacrylate monomer, the depolymerization of various conventional polymethacrylates is successfully demonstrated, thus significantly expanding the depolymerization scope.

Initially, we envisioned that poly­(*O*-aryl methacrylates)^42^ may be excellent candidates to avoid lactonization, thus leading to higher depolymerization under solvent-free conditions. As a model material, poly­(phenyl methacrylate) (PPhMA-Cl) was selected. Poly­(benzyl methacrylate) (PBzMA-Cl) was also chosen as a control polymer, as it possesses an identical structure, except for the presence or absence of benzylic methylene protons (COOCH_2_R), which have previously been reported to play an integral role in the lactonization mechanism (Figure 1a,b).^43,44^ Both polymers were synthesized by activators regenerated electron transfer (ARGET) ATRP, yielding products with comparably high livingness and low dispersity (PBzMA-Cl: livingness = 94% based on chain extension experiment,^45^ *Đ* = 1.16 and PPhMA-Cl: *Đ* = 1.18, Schemes S1 and S2, Figures S1–S6). The catalyst-free bulk depolymerization for both polymers was performed by heating the polymers alone in a TGA (thermal gravimetric analysis) instrument under a nitrogen atmosphere with a dynamic heating rate of 1 °C/min from 25 °C to 450 °C. A distinct difference immediately became apparent between the two polymers, with notably lower temperatures required for mass loss to be observed for the PPhMA. While the onset of mass loss for the PBzMA was 330 °C, and a temperature of 360 °C was required to reach 80% mass loss (Figure 1c, black), for PPhMA, a much lower onset of 230 °C was observed, while only 250 °C was required to reach 80% of mass loss (Figure 1c, green). This indicated that a successful low-temperature bulk depolymerization had occurred for PPhMA in the absence of a catalyst. To confirm that this mass loss corresponded to monomer production, an additional experiment was performed and sampled at 230 °C for ^1^H NMR spectroscopy, whereby distinct peaks corresponding to phenyl methacrylate (PhMA) could be successfully observed (Figure S7). lsothermal reactions were performed for 5 h at 230 °C, taking each polymer in turn. While negligible mass loss was observed for the PBzMA (4%, Figure 1d, black), as much as 86% of the starting monomer could be recovered for the PPhMA (Figure 1d, green). This corresponded to a depolymerization efficiency of 92% and confirmed not only that low-temperature depolymerization was successfully occurring in bulk, but also that near-quantitative monomer yields could be achieved. In fact, this was comparable to an experiment performed with CuCl_2_/TPMA as the catalyst (86% without catalyst vs 84% with catalyst) thus suggesting that the addition of the catalyst does not further improve the percentage of regenerated monomer (Figure S8).

**Figure 1:**
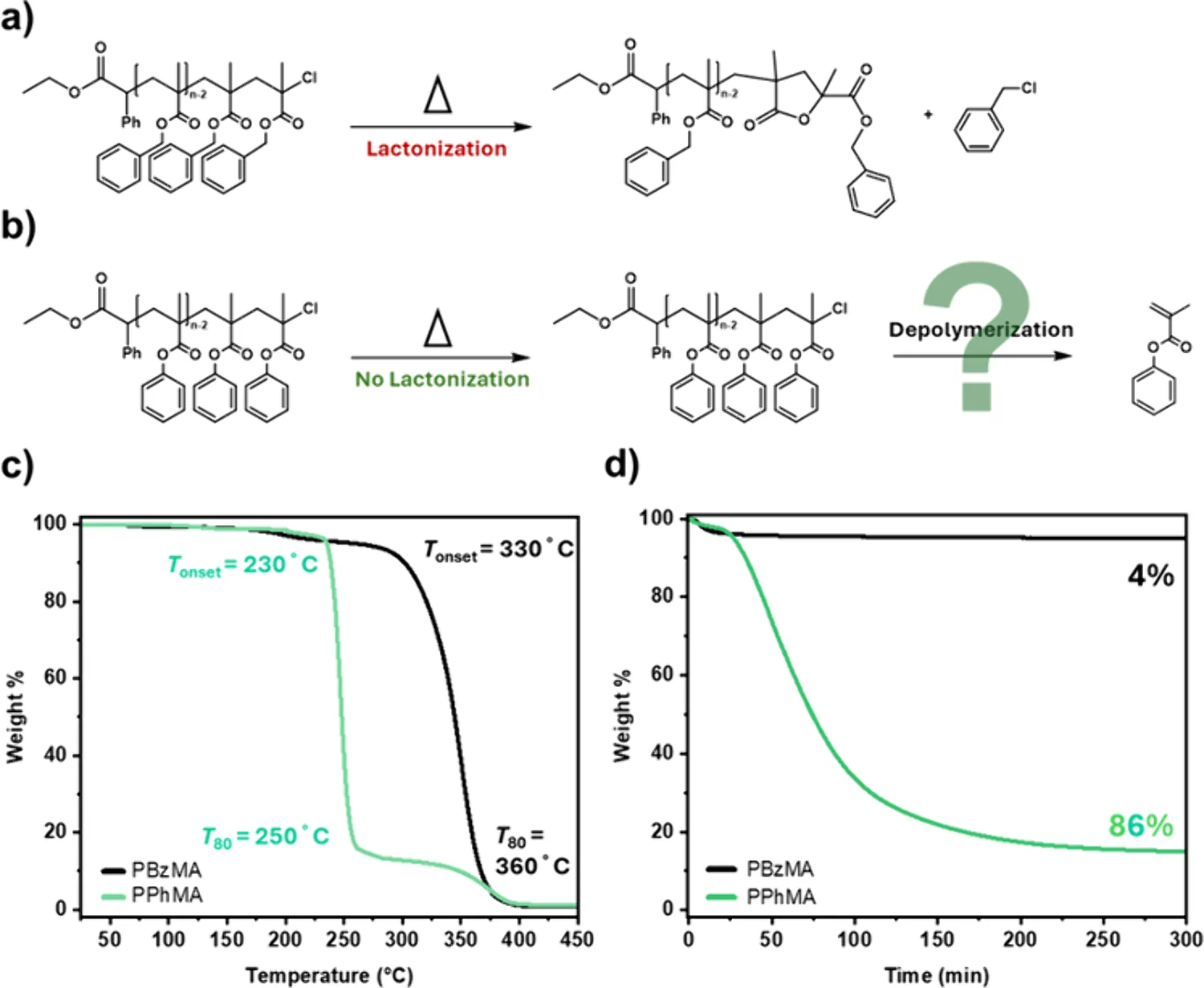
Comparison of the depolymerization of PBzMA and PPhMA in the absence of catalyst. Presented are schemes demonstrating our hypothesis about lactonization for (a) PBzMA and (b) PPhMA. (c) Dynamic and (d) isothermal TGA data are shown for both polymers. Dynamic measurements were performed with a 1 °C/min heating rate between 25 and 450 °C and isothermal depolymerization measurements were carried out at 230 °C for 5 h.

To understand why PPhMA was depolymerizing at lower temperatures, the extent of lactonization for the two polymers after heating them alone to 230 °C for 15 min was investigated. For PBzMA, the formation of benzyl chloride was observed by ^1^H NMR, suggesting that lactonization could occur even over this very short reaction time (Figure S9). To quantify the effect, CuCl_2_/TPMA catalyst was added to this reaction and an additional depolymerization step was performed. This resulted in only a negligible mass loss (6%), which was far lower than the 68% previously reported without the incubation,^41^ illustrating that the polymer chains had been almost completely lactonized and no longer had chlorine end-groups capable of triggering depolymerization (Scheme S3). Interestingly, comparable results were obtained when the polymer incubation time was reduced to just 5 min, suggesting that the lactonization reaction was incredibly fast and providing thermal stability to the polymer (Scheme S4, Figure S10). This contrasted the experimental data for PPhMA, where 86% of depolymerization could be achieved in the absence of catalyst, suggesting lactonization or other side reactions were not occurring (Scheme S5, Figure S11). Together these results confirmed that low-temperature, catalyst-free bulk depolymerization of PBzMA is not feasible and that a catalyst is essential to rapidly activate the polymer chain-ends and to enable depolymerization to outcompete lactonization. In contrast, the absence of benzylic protons in PPhMA prevents lactonization, and stabilizes the chain-ends, paving the way for a catalyst-free depolymerization under bulk conditions. This, nevertheless, raised the important question of how the polymer might be activated in the absence of catalyst (Figure 2a). The possibility of direct homolytic cleavage of the C–Cl bond is unlikely at 230 °C, given the strength of this bond.^46^ Therefore, to investigate further, the dynamic TGA measurement of PPhMA was repeated three times and sampled before, during and after depolymerization (at 200, 245, and 305 °C), for both size exclusion chromatography (SEC) and ^1^H NMR analysis. The three reactions yielded 2%, 50%, and 86% of mass loss, respectively, and on SEC analysis, the samples all had comparable molecular weight distributions and molecular weights (i.e. *M*_n_ = 4600, 4500, 4000), confirming that, upon activation, each polymer chain fully unzips back to monomer (Figure 2b). Subsequently, ^1^H NMR analysis was performed at the same time points. At 200 °C, the spectrum was comparable to that of the starting polymer, albeit with a tiny amount of monomer generation (<1%, peaks at 5.8 and 6.4 ppm, respectively, Figure 2c). However, after sampling the reaction at 245 °C, not only could more significant monomer generation be observed, but two additional low-intensity peaks were present in the vinyl region (5.9 and 6.6 ppm, Figure 2c). The importance of these vinyl peaks became clearer in the sample taken after the depolymerization was complete, i.e., after heating the polymer to 305 °C, as these peaks were no longer present. This disappearance suggested that these vinyl species had formed and then reacted during the depolymerization, and these intermediate species were aiding the depolymerization (Figure 2c). We hypothesized that the peaks corresponded to macromonomer, i.e., vinyl terminal polymer chains, which had previously been reported to facilitate depolymerization at similar temperatures.^37,47^ To confirm this, a low molecular weight PPhMA macromonomer was synthesized by CCTP. On analysis of the product, identical chemical shifts were observed to those found in the depolymerization reactions (5.9 and 6.6 ppm), confirming the formation and subsequent reaction of macromonomer during the catalyst-free bulk depolymerization (Figure S12). Altogether, this demonstrates that in the absence of lactonization, PPhMA prepared by ATRP can be directly depolymerized in the absence of catalyst, most likely via an elimination reaction, which results in the efficient formation of macromonomer (Figure 2a). This in-situ transformation avoids the need for a CCTP modification step and subsequent purification prior to depolymerization.^37^ Further experiments showed that macromonomer formation and depolymerization could also occur at 200 °C (52%), while at temperatures lower than 200 °C, macromonomer did not form and depolymerization was not observed after 8 h (Figures S13 and S14), thus confirming the necessity of the macromonomer formation prior to a successful depolymerization. The temperature required for the reaction also explains why this catalyst-free depolymerization can only be achieved in bulk and not at the lower solution depolymerization temperatures (≤170 °C, Figures S15–S20 and Schemes S6–S9).

**Figure 2:**
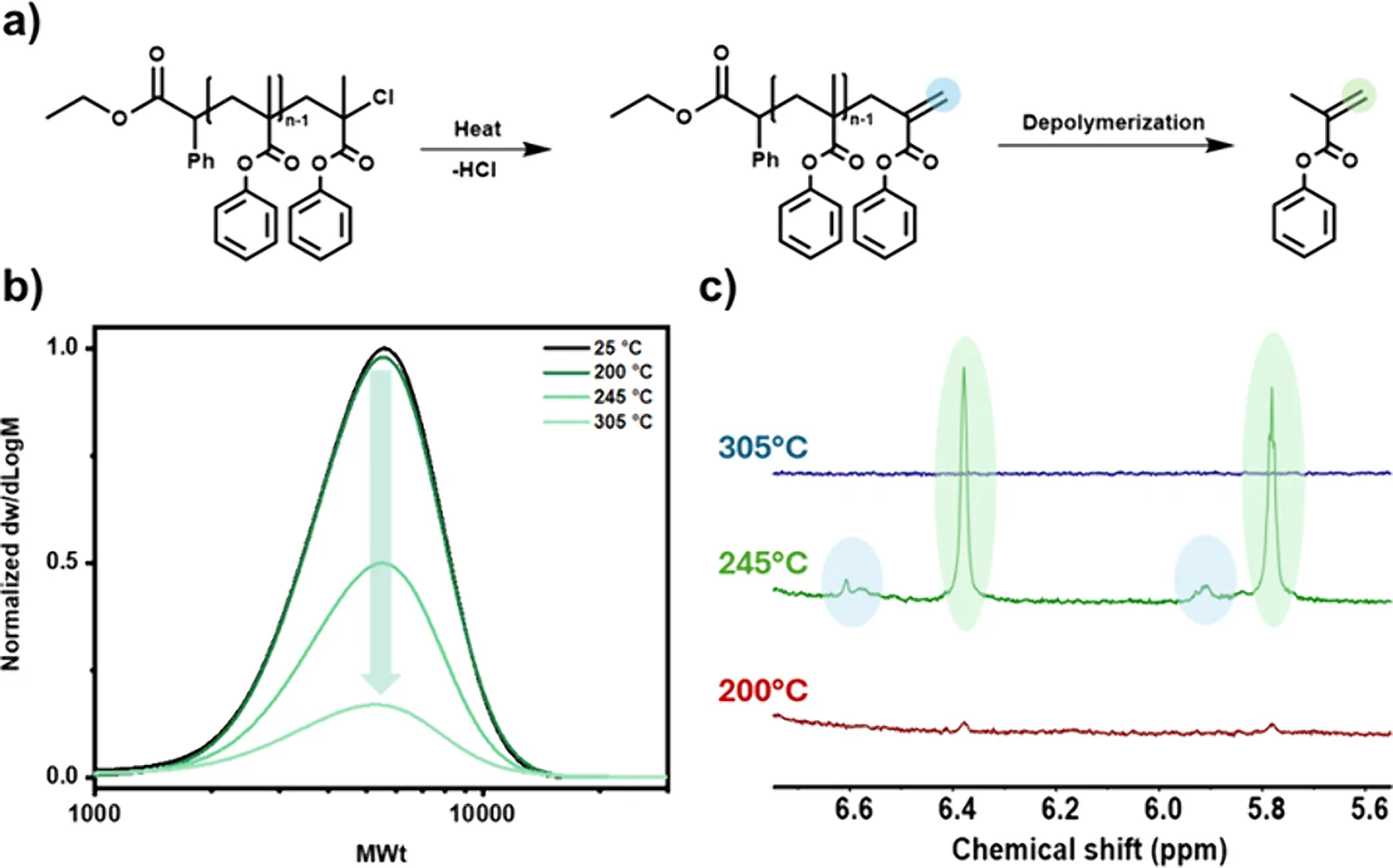
Mechanism of the depolymerization of PPhMA in the absence of catalyst. Presented are (a) a scheme showing the depolymerization pathway, and (b) SEC and (c) ^1^H NMR are shown for samples taken at 200, 245, and 305 °C during the dynamic temperature sweep.

To expand the scope of this catalyst-free depolymerization method, other polymers were synthesized that had the *O*-aryl substitution and would theoretically also avoid lactonization. First, poly­(cresol methacrylate) (PCrMA-Cl) was prepared by ARGET-ATRP with a final *M*_n_ of 7200 and a low dispersity of 1.20. A catalyst-free depolymerization experiment was then performed in bulk at 230 °C. Pleasingly, a rapid mass loss was observed with 80% achieved in 1 h and 88% after 5 h (Figure 3a). To confirm, that monomer was being produced, an additional experiment was performed where the reaction was stopped after 1 h and sampled for ^1^H NMR. The regeneration of cresol methacrylate could clearly be observed, confirming an effective depolymerization process (Figure 3b). Subsequently, poly­(guaiacol methacrylate) (PGuMA-Cl) was also synthesized (*M*_n_ = 6800, *Đ* = 1.21) and successful catalyst-free depolymerization was observed with 80% mass loss obtained by TGA (Figure 3c) and monomer formation evidenced by ^1^H NMR (Figure 3d). Collectively, this demonstrates that a high-yielding catalyst-free bulk-depolymerization is possible for a range of aryl-substituted polymethacrylates. In all cases, reactions are highly efficient and high yields of monomer could be regenerated.

**Figure 3:**
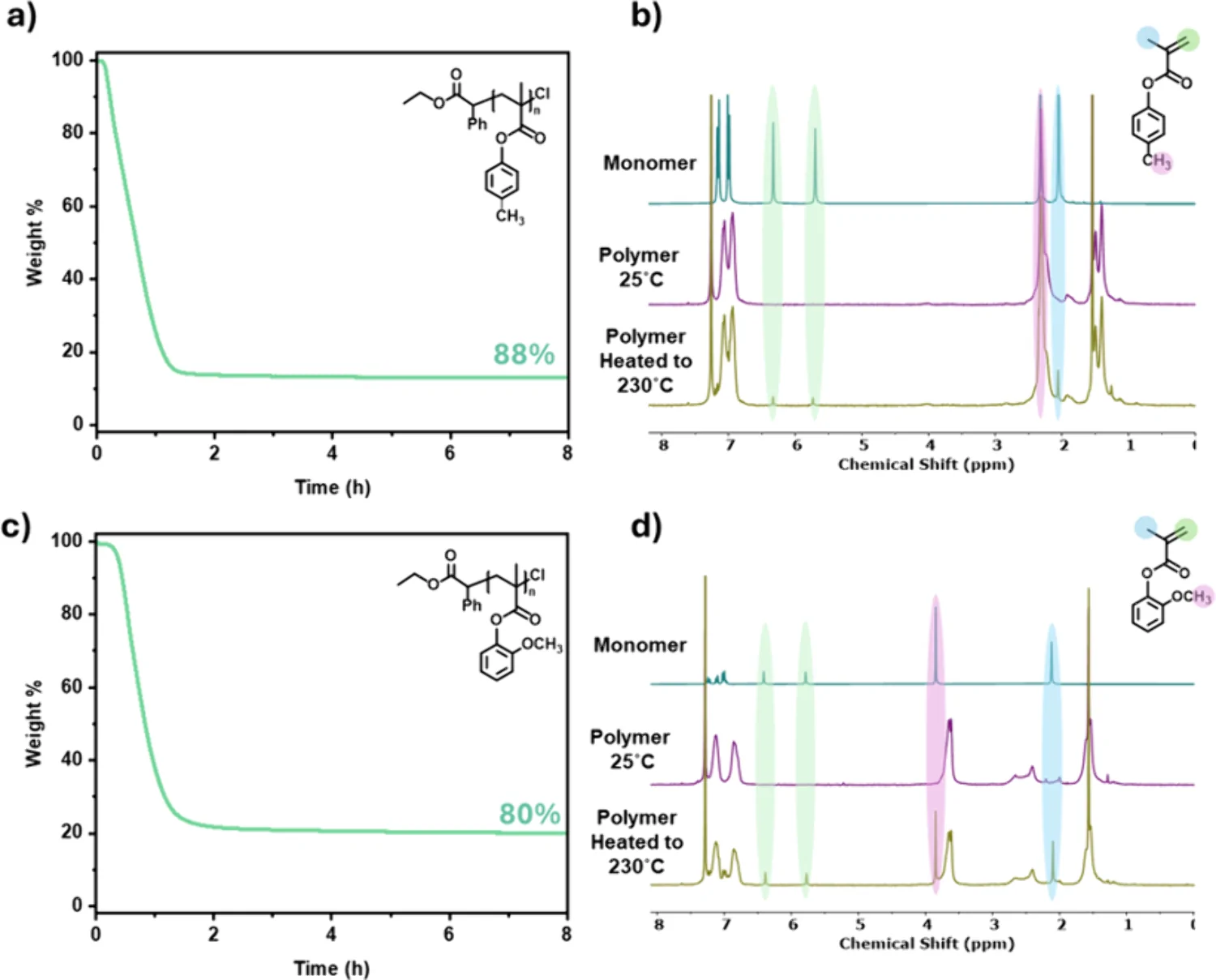
Catalyst-free bulk depolymerization of aryl substituted polymethacrylates at 230 °C. Presented are (a) TGA analysis and (b) ^1^H NMR analysis for the depolymerization of poly­(cresol methacrylate) and (c) TGA analysis and (d) ^1^H NMR analysis for the depolymerization of poly­(guaiacol methacrylate).

To expand the scope of this methodology to conventional methacrylate polymers, we hypothesized that if we installed an *O*-aryl methacrylate monomer on the chain-end of a conventional methacrylate polymer, this would both prevent the lactonization and allow the depolymerization to proceed through the first block. This hypothesis was supported, in the mechanistic section, where the polymer chains had been shown to fully unzip all the way to monomer, suggesting minimal, if any, deactivation. If deactivation is absent, the halogen cannot be transferred back to the polymer chain-end upon activation, thus potentially leading to a higher depolymerization efficiency. To investigate this, PBzMA was chain-extended with a short block of PhMA (Scheme S10) in order to avoid a large percentage of defective chains, as elegantly shown by Harrisson and coworkers.^48^ Pleasingly, successful chain extension was observed, as evidenced by a shift in SEC (*M*_n_ = 6300, *Đ* = 1.10), and the DP of the obtained second block matched that targeted (DP = 6, Figures S21 and S22). Bulk depolymerization was therefore performed at 230 °C, without catalyst, and a high monomer recovery could be successfully achieved (66%, Figure 4a). SEC analysis confirmed that negligible change in the shape of the molecular weight distribution had occurred, indicating the full unzipping of the polymer chains and the generation of both monomers (Figure 4b). The monomer yield was, however, slightly lower than that attained for the poly­(*O*-aryl methacrylate) homopolymers and this was attributed to either a lower livingness of the starting block copolymer or the presence of defective chains, i.e., chains that had not incorporated the phenyl methacrylate monomer during the synthesis. This was confirmed when control experiments were performed with CuCl_2_/TPMA as the catalyst, and an almost identical conversion was obtained (67% vs 66%, Figure S23). To expand the scope further, poly­(methyl methacrylate) (*M*_n_ = 4300, *Đ* = 1.10) was prepared as a second model material and chain extended with phenyl methacrylate to yield a P­(MMA-*b*-PhMA) with an *M*_n_ of 5300 and a dispersity of 1.10 (DP = 8 of PPhMA block, Scheme S11, Figures S24 and S25). On bulk depolymerization, 71% of depolymerization could be achieved alongside a sharp drop in the SEC chromatogram, further validating the effectiveness of the approach (Figure 4c,d). Altogether, this confirms that catalyst-free depolymerization can be applied either directly to poly­(*O*-aryl methacrylates) or to conventional polymethacrylates after a short chain-extension with an *O*-aryl methacrylate. In both cases, monomer recoveries were comparable to those obtained with a copper catalyst.

**Figure 4:**
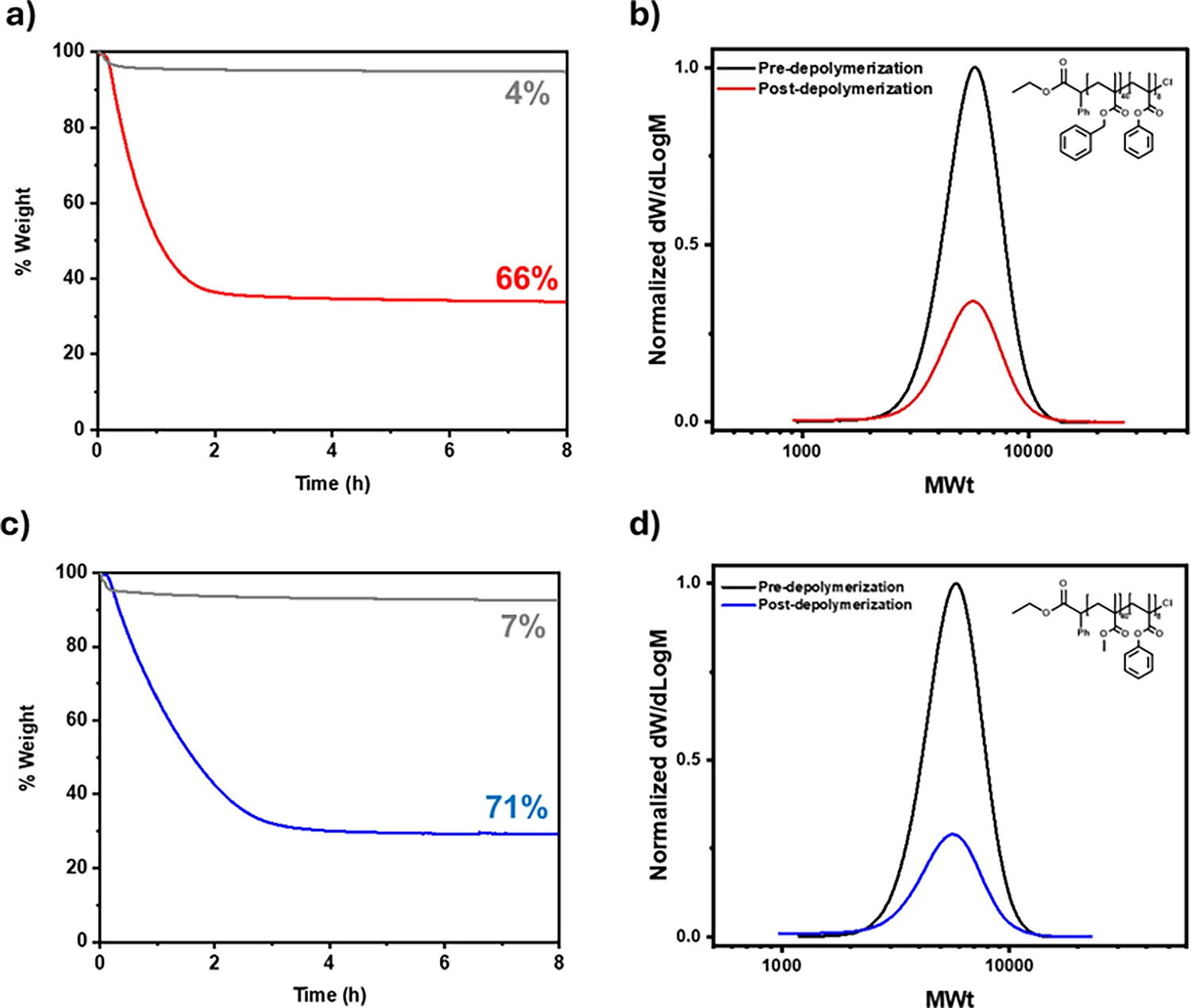
Catalyst-free bulk depolymerization of conventional polymethacrylates at 230 °C. Presented are (a) TGA analysis and (b) SEC analysis for the depolymerization of poly­(benzyl methacrylate) before (black) and after (red) chain extension with phenyl methacrylate and (c) TGA analysis and (d) SEC analysis for the depolymerization of poly­(methyl methacrylate) before (black) and after (blue) chain extension with phenyl methacrylate.

To summarize, the possibility of catalyst-free depolymerization of ATRP polymers has been demonstrated for various polymethacrylates. Specifically, the low-temperature depolymerization of poly­(*O*-aryl-methacrylates) yields high percentages of the starting monomer (∼90%). For conventional methacrylates, lactonization outcompetes depolymerization, preventing monomer recovery, however, on the addition of a small block of an *O*-aryl methacrylate, this problem is overcome and successful depolymerization can be achieved.

## Supplementary Material



## Notes

Experimental section and supporting figures (PDF)
