# Supplementary material for: Catalyst-Free Bulk Depolymerization of Polymethacrylates Prepared by ATRP
Source: ACS Macro Lett. 2026 Sep 10;15(10):1468–74. doi: 10.1021/acsmacrolett.6c00380 (PMC13689910; doi:10.1021/acsmacrolett.6c00380)
Supplement: Supplementary file 1 [file mz6c00380_si_001.pdf]

Supplementary Information

Catalyst-Free Bulk Depolymerization of Polymethacrylates Prepared by ATRP

Brendan Pozzi, Stella Afroditi Mountaki, Richard Whitfield*, Athina Anastasaki*

Laboratory of Sustainable Polymers, Department of Materials, ETH Zurich, Vladimir-Prelog-Weg 5, 8093 Zurich, Switzerland.

Contents Page

Materials and Instrumentation: 3
Methods 4
Methods for Monomer Synthesis 4
Methods for Polymer Synthesis 4
Methods for Depolymerization 6
Additional Characterization Data 8
PBzMA Synthesis 8
PPhMA Synthesis 10
Solution Depolymerization of PBzMA and PPhMA 19
Bulk Depolymerization of PBzMA and PPhMA 12
Mechanism of Catalyst-Free Bulk Depolymerization 14
Catalyst-Free Bulk Depolymerization of Conventional Polymethacrylates 25
References 29

Materials and Instrumentation:

Chemicals were purchased from Sigma-Aldrich, Acros Organics or Tokyo Chemical Industry and unless otherwise specified, used as received. All monomers were passed through a basic alumina column prior to use.

^{1}H NMR spectra were measured on a Bruker DPX-300 spectrometer in deuterated chloroform ($CDCl_3$) or deuterated acetone (acetone-d6). Monomer conversions were calculated by comparing the integral of vinyl protons in the monomer with those from both monomer and polymer signals.

Size Exclusion Chromatography (SEC) analysis was performed using a Shimadzu modular system consisting of a CBM-20A system controller, a SIL-20A automatic injector, a 10.0 μm bead size guard column (50 × 7.5 mm), followed by three KF-805L columns (300 × 8 mm, bead size: 10 μm, pore size maximum: 5000 Å) and an RID-20A differential refractive-index detector. The eluent was N,N-dimethylacetamide (HPLC grade, with 0.03% w/v LiBr) and the flow rate was maintained at 1 mL min^{-1} using an LC-20AD pump. A calibration curve for molecular weight was generated using commercially available narrow molecular weight distribution poly(methyl methacrylate) standards spanning from 5000 to 1.5 × 10^{6} Da. SEC samples were prepared in dimethylacetamide, followed by filtration through a basic alumina column and a 0.45 μm PTFE filter. For depolymerization experiments, normalization of SEC traces was performed based on the depolymerization extent as measured by TGA.

Thermogravimetric Analysis (TGA) was performed on a Mettler Toledo TGA/DSC 3+ stare system equipped with a Mettler Toledo ultra-micro balance. Experiments were performed in 70 μL alumina crucibles with the specified method. Measurements were always carried out under inert atmosphere (100 mL/min N_2 flow rate). Unless stated otherwise, dynamic measurements were performed with a 1˚C/minute heating rate from 25-450˚C and isothermal measurements were carried out with a 50˚C/min initial heating rate before maintaining a temperature of 230˚C for 15 minutes. It is important to note that observed weight loss is not solely attributed to depolymerization; contributions from polymer end-group loss or residual solvent may also be present. To minimize these effects, all polymer samples were thoroughly dried under vacuum prior to analysis.

Methods

Methods for Monomer Synthesis

In a 500 mL round bottom flask equipped with a stir bar, phenol (1 equiv., 0.33 mol, 31.1 g) was dissolved in 2-methyltetrahydrofuran (82 mL) and triethylamine (1.1 equiv., 0.36 mol, 50 mL). Methacrylic anhydride (1.1 equiv., 0.36 mol, 49.2 mL) was then added and the mixture was heated at 40 °C for 16 hours. The reaction mixture was then cooled to room temperature, washed with sat. $NaHCO_3$ (100 mL), dried over Na_2SO_4 and filtered with a cotton plug. Excess solvent was removed in vacuo to leave a yellow crude, which was purified by flash column chromatography (eluent: 9/1 ratio petroleum ether/EtOAc). The collected fractions containing the product were then concentrated to yield the colorless monomer.

Methods for Polymer Synthesis

PBzMA-Cl Synthesis by ARGET-ATRP:

In a 10 mL vial, a solution of $CuCl_2$ (0.05 equiv., 0.012 mmol, 1.6 mg) and PMDETA (0.15 equiv., 0.035 mmol, 7.4 µL) in acetonitrile (200 µL) was prepared. The solution was then added to a 10 mL vial equipped with a stir bar and 4 mls of BzMA (100 equiv., 23.6 mmol), 40.5 µLs of ECPA (1 equiv., 0.24 mmol) and 1 mL of anisole. In another 10 mL vial, a solution of Sn(eh)2 (0.2 equiv., 0.047 mmol, 19.2 mg) and anisole (0.8 mL) was prepared. Both vials were then sealed with a septum and bubbled with N_2 for 15 minutes. The $Sn(eh)_2$ solution was then added to the vial containing the monomer using a syringe flushed with N_2. The mixture was then heated in an oil bath at 70 °C until the desired molecular weight was achieved (80 min). Reaction monitoring was done with a combination of ^{1}H-NMR and SEC analysis based on small reaction mixture aliquots. After the reaction was complete, the vial was opened and cooled to room temperature. The crude was then dissolved in acetone and passed through a basic alumina column. The solution was then concentrated and repeatedly precipitated in cold pentane (4x50 mL) until all monomer was removed. The solids were then collected in a vial and dried in vacuo until the solvent was completely evaporated. The dried polymer was then characterized by SEC and ^{1}H-NMR.

PPhMA-Cl Synthesis by ARGET-ATRP:

Reaction conditions were kept consistent with the synthesis of PBzMA. An identical ratio of [PhMA]:[ECPA]:[$CuCl_2$]:[PMDETA]:[$Sn(eh)_2$] = 100:1:0.05:0.15:0.2, was used. Similarly, the monomer/solvent ratio of 2/1 and the anisole/ACN ratio of 9/1 were kept consistent for the experiments. A similar procedure was used for the synthesis of PCrMA and PGuMA.

Chain Extension to Test Polymer Livingness

Chain extensions were performed analogously to polymerization reactions but with a polymer macroinitiator. A ratio of [M]:[P]:[$CuCl_2$]:[PMDETA]:[$Sn(eh)_2$] = 300:1:0.05:0.15:0.4, was used for the syntheses. The monomer/solvent ratio of 2/1 and the anisole/ACN ratio of 9/1 were kept consistent for the experiments. Reactions were degassed by bubbling with nitrogen and reaction time was 3 hours at 70˚C. The livingness of the polymer was then calculated after deconvolution of the SEC traces.

End-group Modification of PBzMA-Cl by ARGET-ATRP:

End-group modification syntheses were performed analogously to the chain extension reaction, but with a lower ratio of polymer to monomer. Reaction was performed with a ratio of [PBzMA]:[PhMA]:[$CuCl_2$]:[PMDETA]:[$Sn(eh)_2$] = 1:20:0.05:0.15:0.2. The monomer/solvent ratio of 2/1 and the anisole/ACN ratio of 9/1 were kept consistent for the experiments.

Reactions were degassed by bubbling with nitrogen and reaction time was 30 minutes at 70˚C. After reaction was complete, the crude reaction mixture was dissolved in acetone, passed through a basic alumina column, concentrated and repeatedly precipitated in cold pentane until all monomer was removed. The solids were then collected in a vial and dried in vacuo. The dried polymer was then characterized by SEC and ^{1}H-NMR.

End-group Modification of PMMA-Cl by ARGET-ATRP:

Reaction conditions were kept consistent with the synthesis of P(BzMA)-PhMA-Cl. A ratio of [PMMA]:[PhMA]:[$CuCl_2$]:[PMDETA]:[$Sn(eh)_2$] = 1:20:0.05:0.15:0.2. was used. Similarly, the monomer/solvent ratio of 2/1 and the anisole/ACN ratio of 9/1 were kept consistent for the experiments.

CCTP of PhMA to prepare PPhMA Macromonomer

2 mg of CoBF was weighed into a 20 mL glass vial. It was sealed with a septum and was flushed with nitrogen for 20 minutes. In parallel, 10 ml of toluene was added to a 20 mL glass vial. This was also sealed with a septum and bubbled with nitrogen for 20 minutes. A gas-tight syringe was purged with nitrogen 3 times and used to transfer all 10 mL of toluene into the vial with the catalyst. This was then vortexed until full dissolution had occurred. In a 5 mL vial, 7.6 mg of AIBN (0.01 equiv.), 0.5 mL of phenyl methacrylate (1 equiv.), 0.5 mL of toluene and a magnetic stirrer bar were added. This was also sealed with a septum and bubbled with nitrogen for 20 minutes. Finally, a gas-tight syringe was purged with nitrogen 3 times and used to transfer 0.5 mL of catalyst solution (0.1 mg of CoBF) into the vial with the monomer, initiator and solvent. The reaction was then placed in an oil bath at 70˚C on a magnetic stirrer hot plate. Reaction was performed for 18 hours. After the reaction was complete, the vial was removed from the heat and opened to air. It was sampled for ^{1}H NMR analysis.

Methods for Depolymerization

Attempted Catalyst-free Solution Depolymerization of PBzMA

In a 5 mL vial, 9.1 mgs of PBzMA (1 equiv. $[RU]_0$= 50mM, 1.4 x 10^{-3} mmol) and 4.6 mgs of a polystyrene standard were dissolved in 1 mL of 1,2,4-trichlorobenzene. In another 5 mL vial, the catalyst stock solution was prepared by dissolving 2 mgs of $CuCl_2$ and 35.7 mgs of TPMA in 140 µL of DMF. Then, 2 µL of the stock solution containing 0.04 mgs of $CuCl_2$ (0.22 equiv., 3 x 10^{-4} mmol) and 0.51 mgs of TPMA (1.3 equiv., 1.8 x 10^{-3} mmol) were added to the PBzMA solution. A stirrer bar was added, and then it was stirred vigorously to dissolve all solids before being sealed with a rubber septum. The solution was then bubbled for 15 minutes with nitrogen, a small aliquot was then collected (t = 0) with a syringe flushed with nitrogen. The mixture was then heated at 170 °C for 15 minutes, after which the test tube was cooled to room temperature in a water bath and subsequently opened to air. The depolymerization yields were then determined by ^{1}H NMR with the integration of the monomer vinyl peaks or by SEC with the comparison of the polymer peak intensity relative to the polystyrene standard.

Attempted Catalyst-free Solution Depolymerization of PPhMA

An analogous experiment was performed as for the depolymerization of PBzMA. More specifically, reactions were performed with a $[RU]_0$ = 50 mM in 1 mL of TCB and 0.2 µL of DMF at 170 °C for 15 minutes.

Incubation Solution Depolymerization Experiments

Incubation experiments were performed for 24 hours in the absence of catalyst and subsequently 0.22 equivalents of $CuCl_2$ and 1.3 equivalents of TPMA were added with a degassed syringe from a degassed stock solution. Depolymerization was then allowed to proceed for a further 15 minutes at 170 °C, prior to sampling for ^{1}H NMR and SEC analysis

Attempted Catalyst-free Bulk Depolymerization of PBzMA

Between 6 and 8 mg of PBzMA-Cl were added to a pre-weighed, cleaned and burned, 70 *µ*L alumina crucible. The crucible was then reweighed and the mass of the sample was recorded. The sample was then placed into a TGA under an inert atmosphere (50 mL/min of nitrogen gas) and heated at 50˚C/minute until a temperature of 230˚C was reached. The reaction was then allowed to proceed for 15 minutes at this temperature. Once the reaction was complete, the crucible was removed from the TGA and collected for subsequent ^{1}H NMR or SEC analysis. ^{1}H NMR analysis was performed on dissolution of the sample in $CDCl_3$, while SEC analysis was performed on dissolution in DMAc. All samples were filtered through a column of basic alumina and a 0.45µM PTFE filter before SEC analysis.

Dynamic measurements were performed by heating between 6 and 8 mg of the polymer from 25˚C to 450˚C with a 1˚C/minute heating rate.

Catalyzed Bulk Depolymerization of PBzMA

In a 5 mL glass vial, 3.8 mgs of $CuCl_2$ and 8.1 mgs of TPMA were dissolved in 2 mL of acetone. In a separate 5 mL glass vial, 8 mgs of PBzMA (1 equiv.) were weighed and dissolved in acetone. 20 µL of the $CuCl_2$/TPMA mixture was subsequently added to the polymer and the sample was dried under a flow of nitrogen to a volume of around 50 µL. The 50 µL was then transferred to a pre-weighed, cleaned and burned, 70 *µ*L alumina crucible and dried under a flow of nitrogen and then under vacuum, until a consistent mass was reached. The crucible was then reweighed and the mass of the sample was recorded. The sample was then placed into a TGA under an inert atmosphere (50 mL/min of nitrogen gas) and heated at 50˚C/minute

until a temperature of 230˚C was reached. The reaction was then allowed to proceed for 15 minutes at this temperature. Once the reaction was complete, the crucible was removed from the TGA and collected for subsequent ^{1}H NMR or SEC analysis. ^{1}H NMR analysis was performed on dissolution of the sample in $CDCl_3$, while SEC analysis was performed on dissolution in DMAc. All samples were filtered through a column of basic alumina and a 0.45μM PTFE filter prior to SEC analysis.

Incubation experiments were performed for 5 or 15 minutes in the absence of catalyst and subsequently the product of the reaction was dissolved in acetone and the requisite amount of $CuCl_2$/TPMA solution added prior to an identical drying and weighing procedure. Reactions were performed for 15 minutes at 230˚C.

Catalyst-free Bulk Depolymerization of PPhMA

Between 6 and 8 mgs of PPhMA-Cl was added to a pre-weighed, cleaned and burned, 70 *μ*L alumina crucible. The crucible was then reweighed and the mass of the sample was recorded. The sample was then placed into a TGA under an inert atmosphere (50 mL/min of nitrogen gas) and heated at 50˚C/minute until a temperature of 230˚C was reached. The reaction was then allowed to proceed for 15 minutes at this temperature. Once the reaction was complete, the crucible was removed from the TGA and collected for subsequent ^{1}H NMR or SEC analysis. 1H NMR analysis was performed on dissolution of the sample in $CDCl_3$, while SEC analysis was performed on dissolution in DMAc. All samples were filtered through a column of basic alumina and a 0.45μM PTFE filter prior to SEC analysis. An identical procedure was used for the depolymerization of PCrMA and PGuMA.

Catalyst-free Bulk Depolymerization of P(BzMA-b-PhMA)

Between 6 and 8 mg of P(BzMA-b-PhMA-Cl) was added to a pre-weighed, cleaned and burned, 70 *μ*L alumina crucible. The crucible was then reweighed and the mass of the sample was recorded. The sample was then placed into a TGA under an inert atmosphere (50 mL/min of nitrogen gas) and heated at 50˚C/minute until a temperature of 230˚C was reached. The reaction was then allowed to proceed for 15 minutes at this temperature. Once the reaction was complete, the crucible was removed from the TGA and collected for subsequent ^{1}H NMR or SEC analysis. ^{1}H NMR analysis was performed on dissolution of the sample in $CDCl_3$, while SEC analysis was performed on dissolution in DMAc. All samples were filtered through a column of basic alumina and a 0.45μM PTFE filter prior to SEC analysis. An identical procedure was used for the depolymerization of P(MMA-b-PhMA)

Additional Characterization Data

PBzMA Synthesis

Scheme S1: Scheme illustrating the synthesis of PBzMA via ARGET ATRP. A ratio of [ECPA]:[BzMA]:[$CuCl_2$]:[PMDETA]:[$Sn(eh)_2$] = 1:100:0.1:0.15:0.2 was used with anisole as the solvent and a temperature of 70˚C.

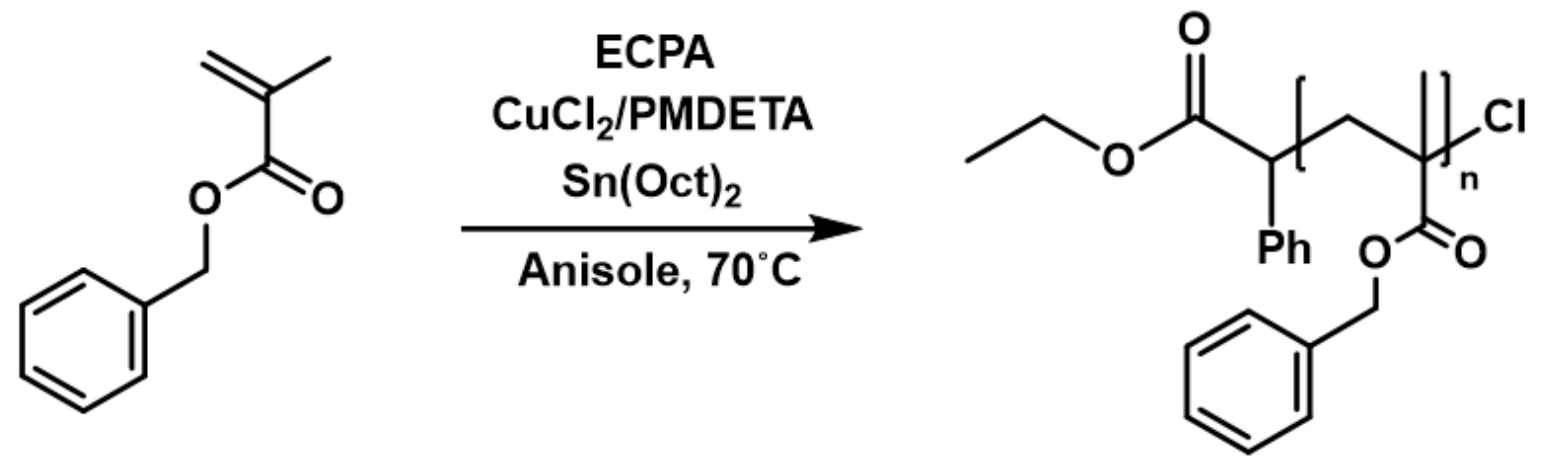

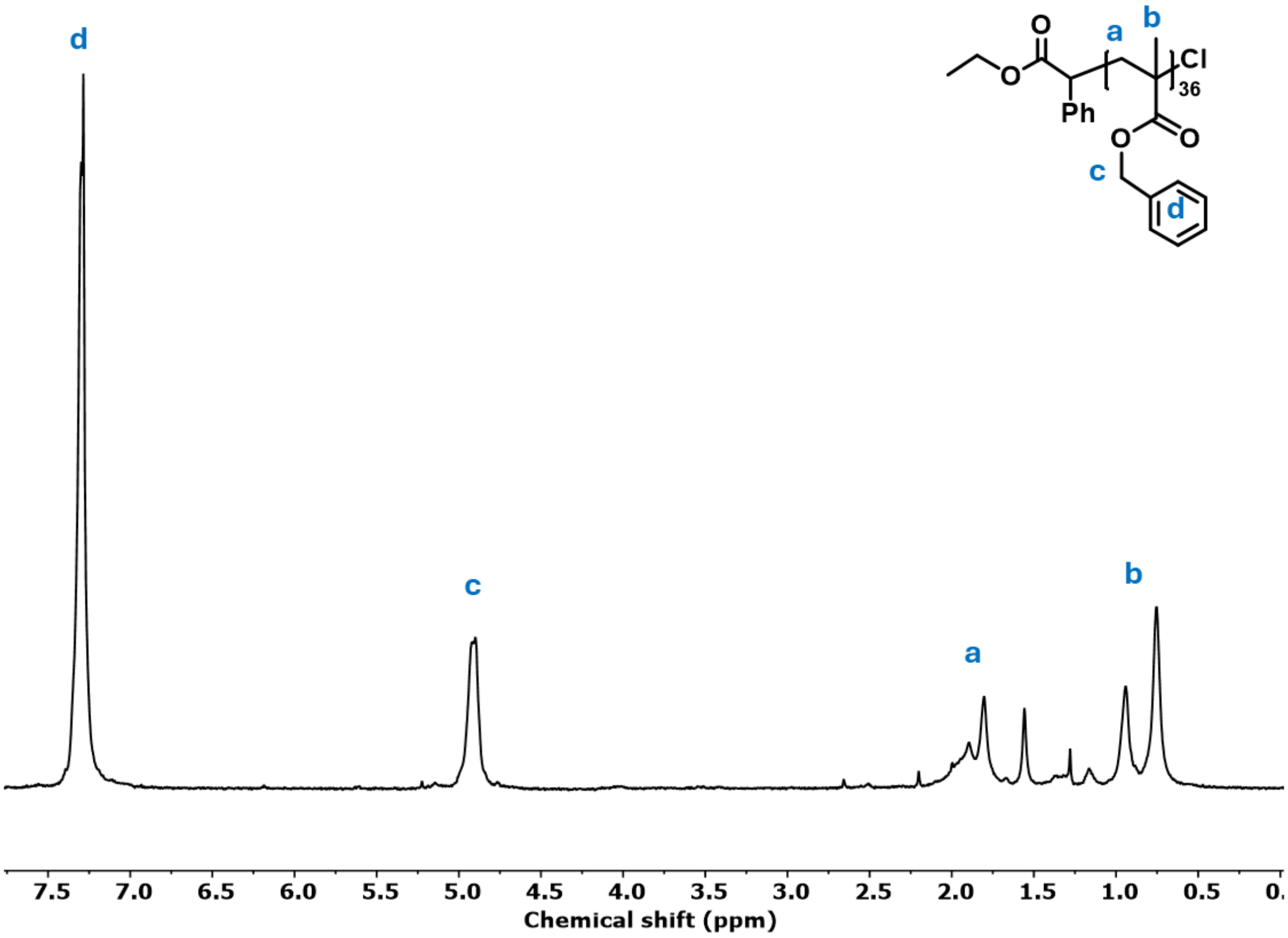

Figure S1: ^{1}H NMR of the purified PBzMA prepared by ATRP.

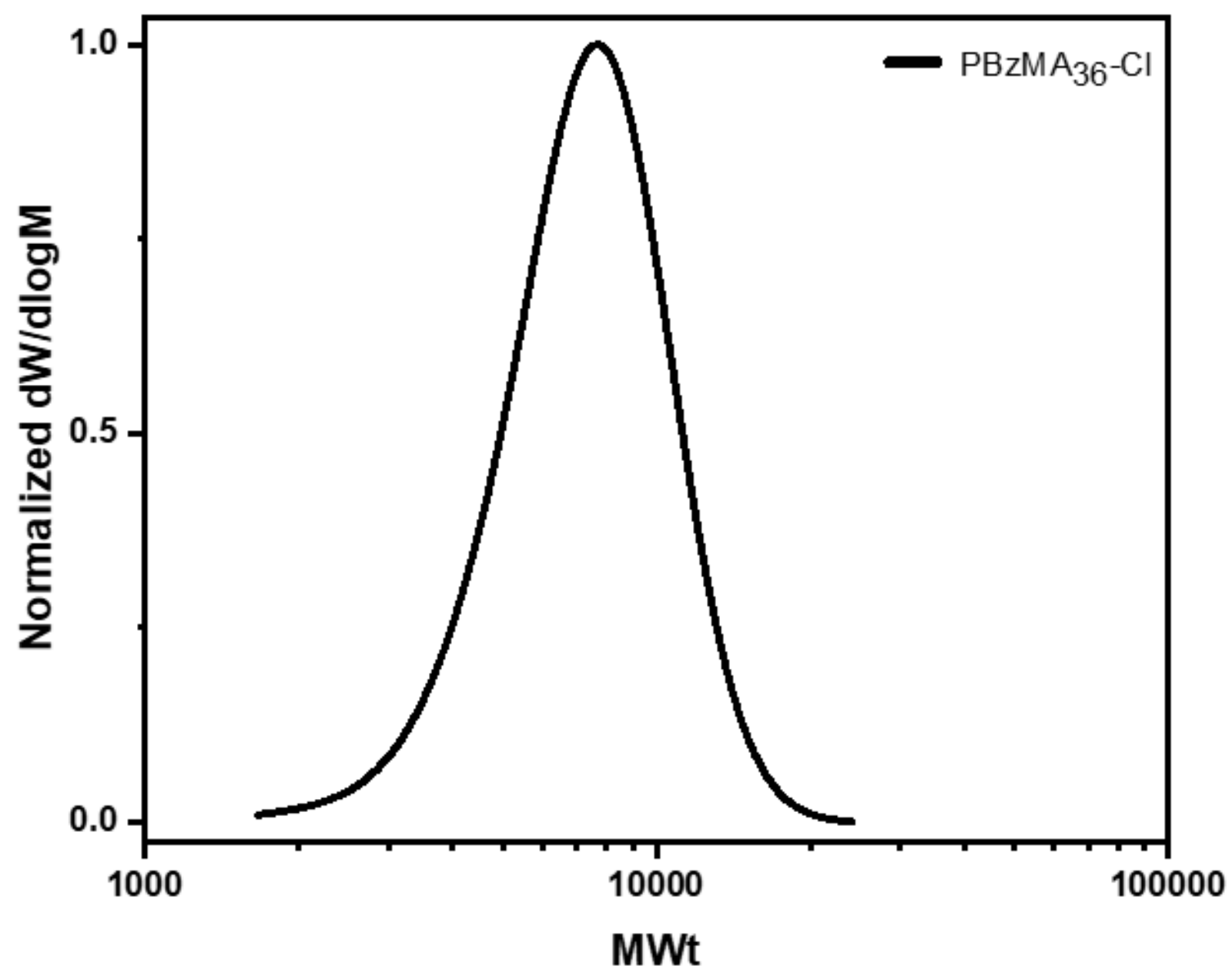

Figure S2: SEC trace of the purified PBzMA prepared by ATRP. The M_n of this polymer is 6300 and the dispersity is 1.16.

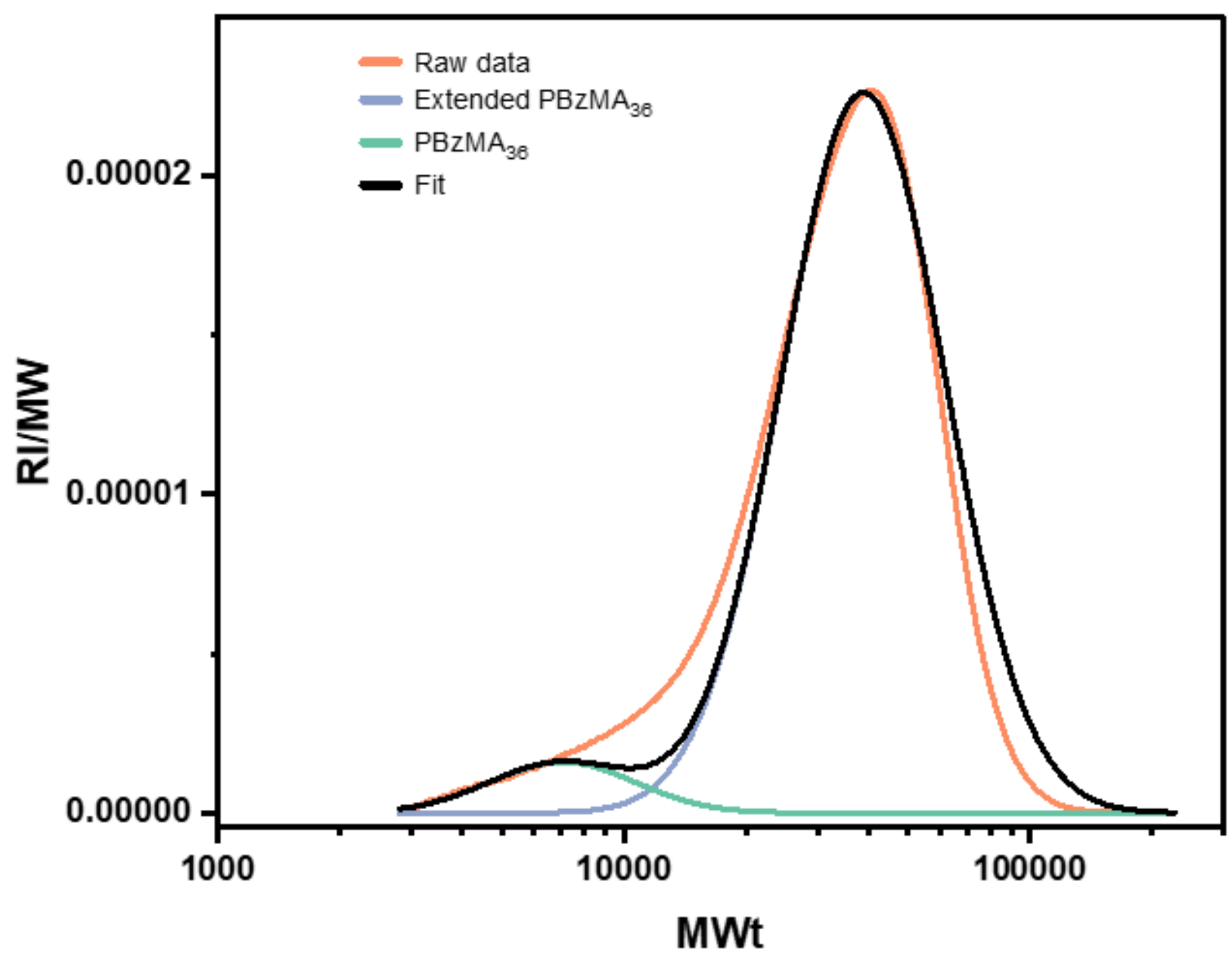

Figure S3: Deconvoluted SEC traces of chain extended PBzMA-Cl to quantify livingness. The livingness was calculated to be 94% based on the comparison of the integration area of the extended P(BzMA-*b*-BzMA)-Cl versus the unreacted PBzMA-Cl. This was based on the methodology reported by Wooley et al.[1]

PPhMA Synthesis

Scheme S2: Scheme illustrating the synthesis of PPhMA via ARGET ATRP. A ratio of [ECPA]:[PhMA]:[$CuCl_2$]:[PMDETA]:[$Sn(eh)_2$] = 1:100:0.1:0.15:0.2 was used with anisole as the solvent and a temperature of 70˚C.

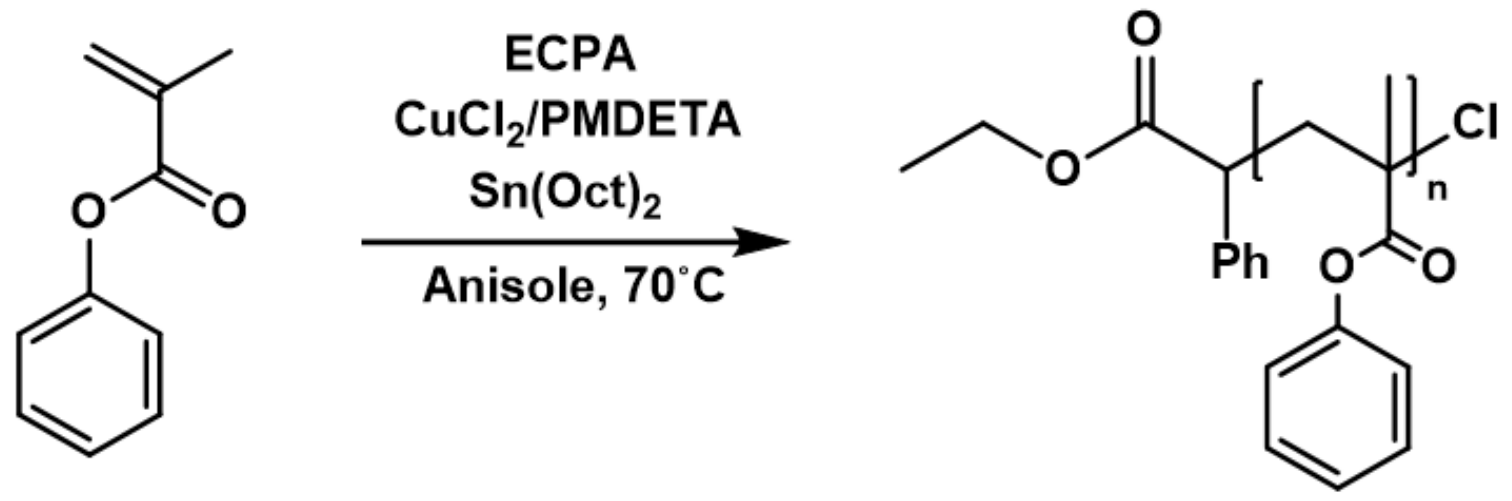

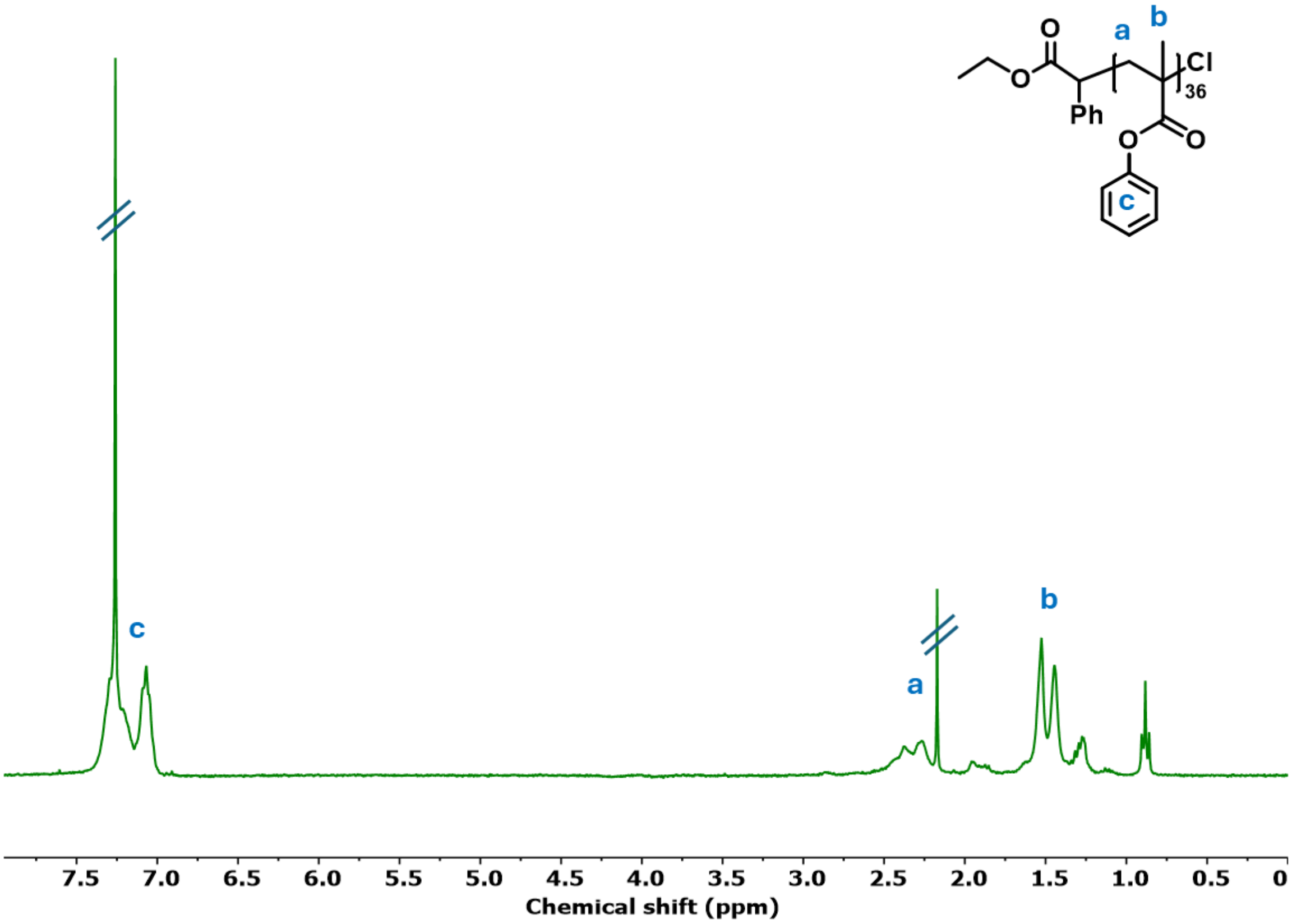

Figure S4: ^{1}H NMR of the purified PPhMA prepared by ATRP.

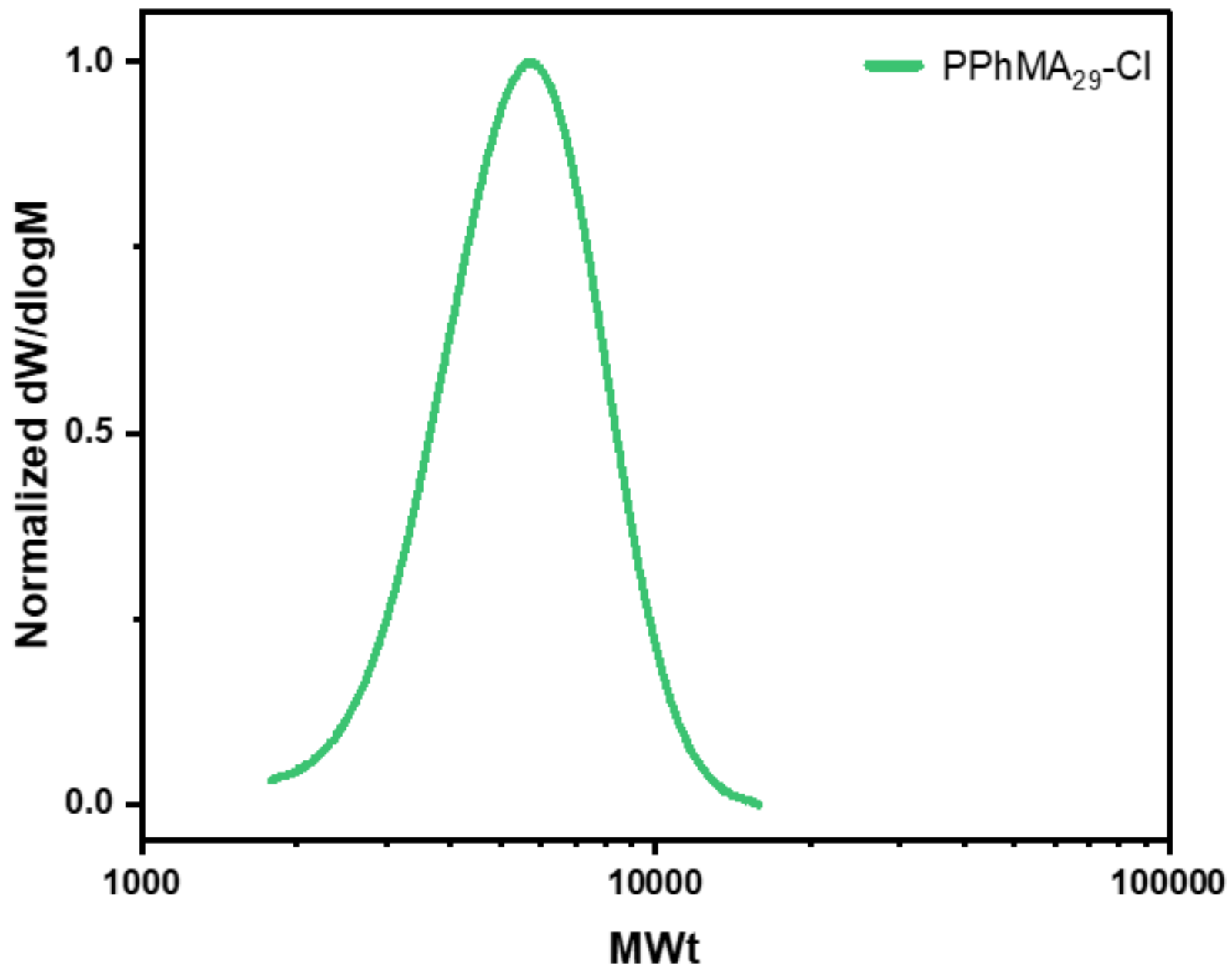

Figure S5: SEC trace of the purified PPhMA prepared by ATRP. The M_n of this polymer is 5000 and the dispersity is 1.16.

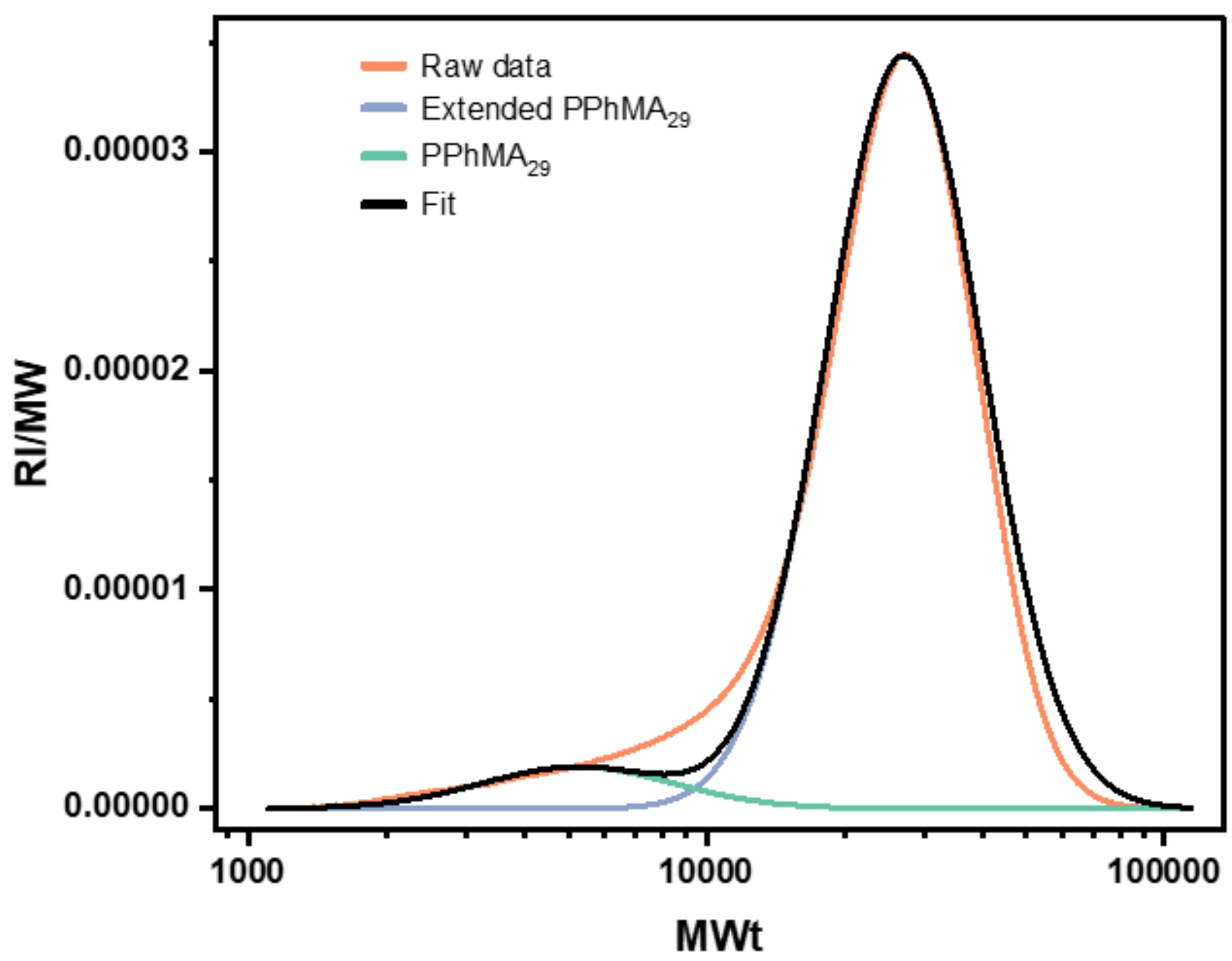

Figure S6: Deconvoluted SEC traces of chain extended PPhMA-Cl to quantify livingness. The livingness was calculated to be 94% based on the comparison of the integration area of the extended P(PhMA-*b*-PhMA)-Cl versus the unreacted PPhMA-Cl.

Bulk Depolymerization of PBzMA and PPhMA

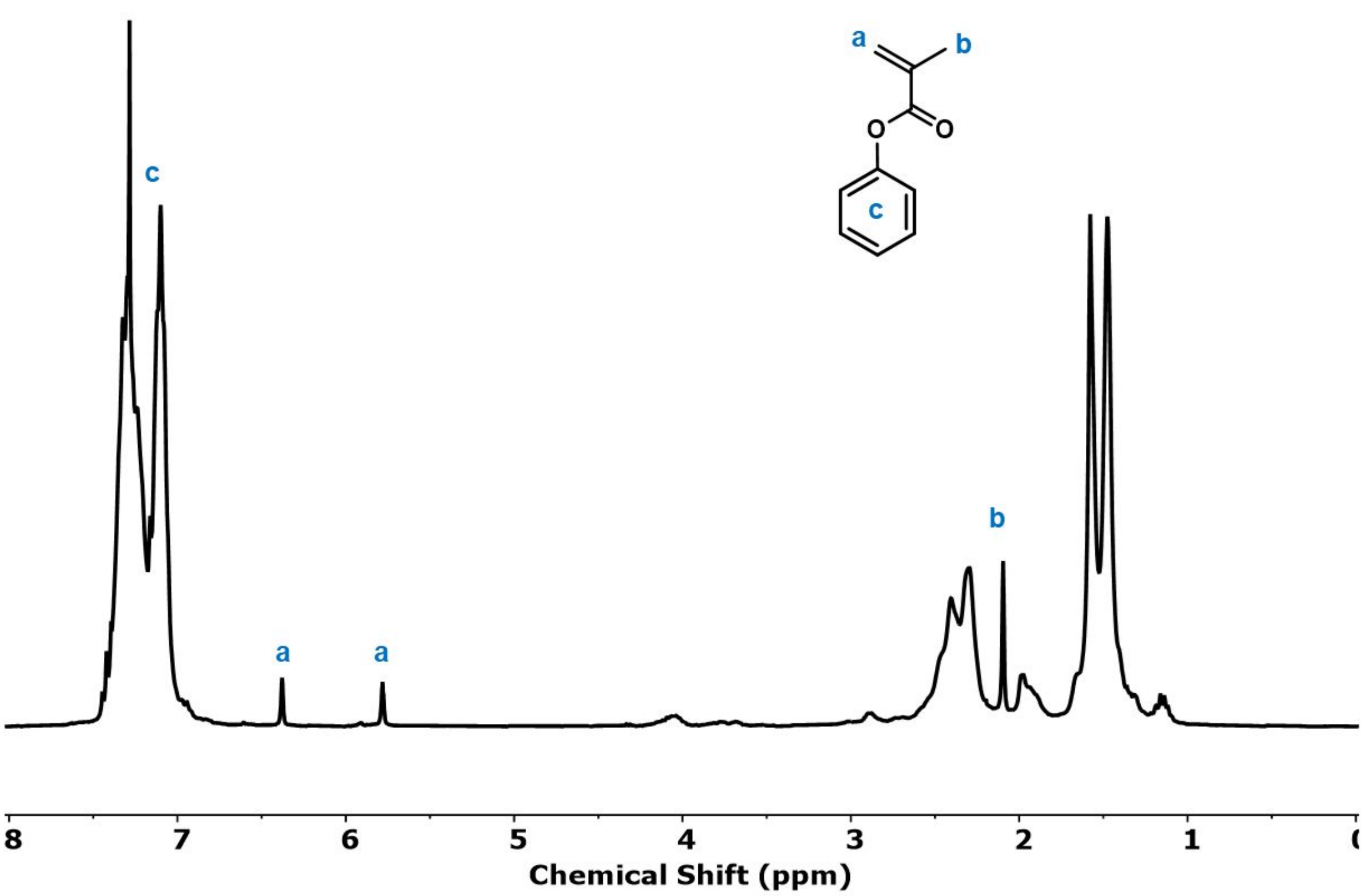

Figure S7: ^{1}H NMR obtained during the catalyst-free bulk depolymerization of PPhMA. Reaction was performed at 230˚C and the presence of monomer is observed and labelled.

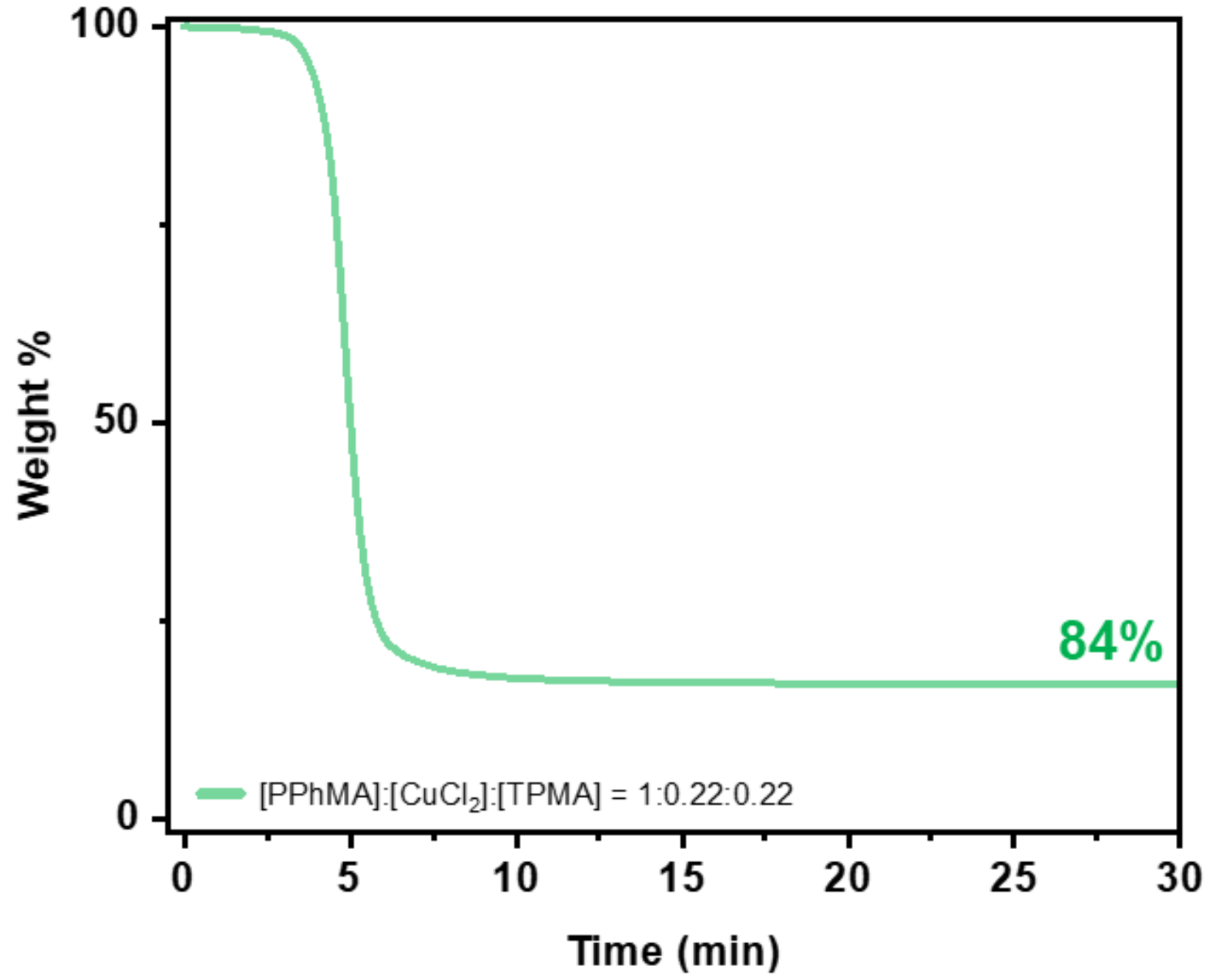

Figure S8: TGA analysis for the $CuCl_2$/TPMA catalyzed depolymerization of PPhMA. The conversion was 84% compared to 86% for the catalyst-free reaction. Reaction was performed at 230˚C with a ratio of [PPhMA]:[$CuCl_2$]:[TPMA]=1:0.22:0.22.

Mechanism of Catalyst-Free Bulk Depolymerization

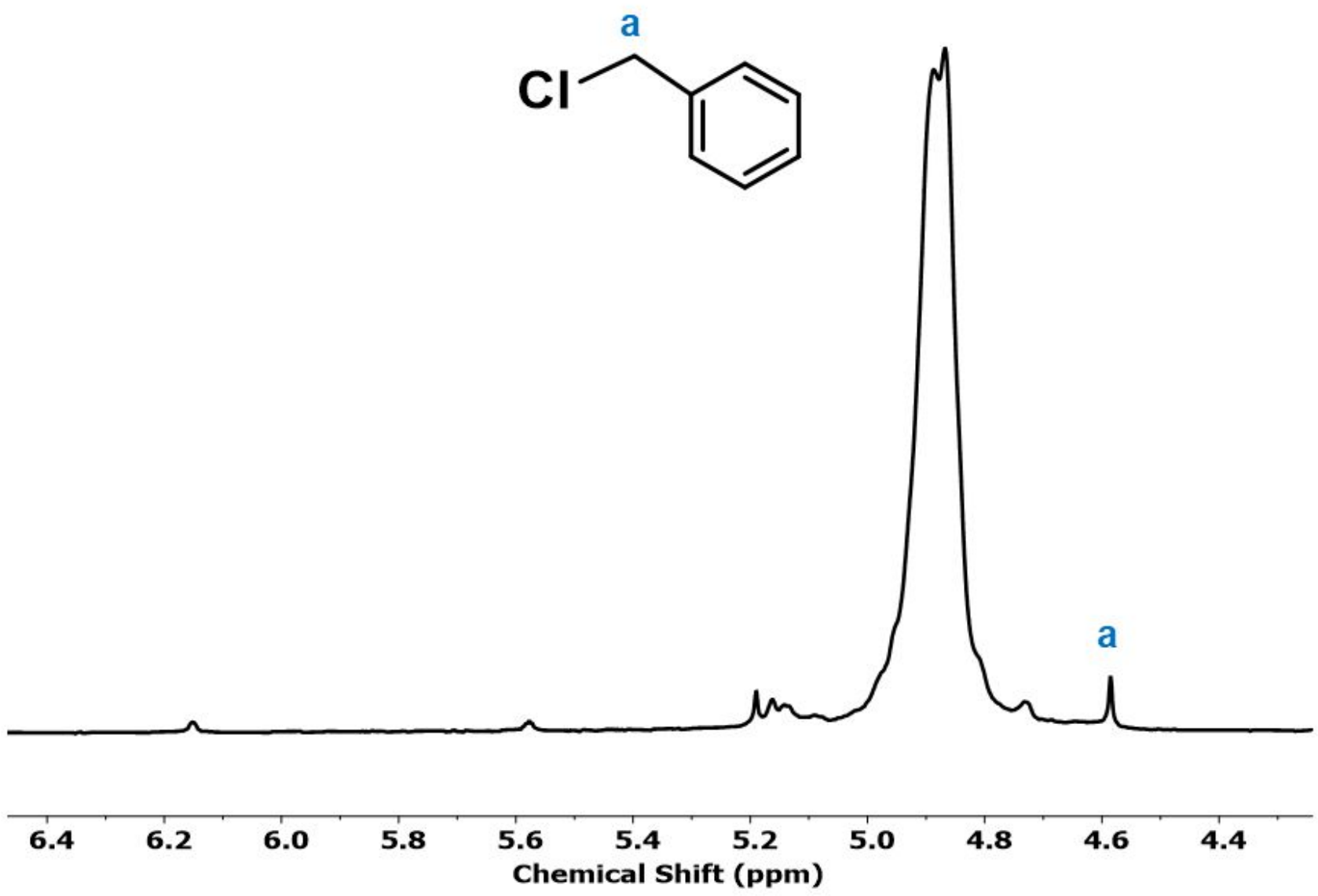

Figure S9: ^{1}H NMR demonstrating the formation of BzMA-Cl on heating PBzMA to 230˚C.

Scheme S3: Copper catalyzed bulk depolymerization of PBzMA after a 15-minute incubation of the polymer in the absence of catalyst.

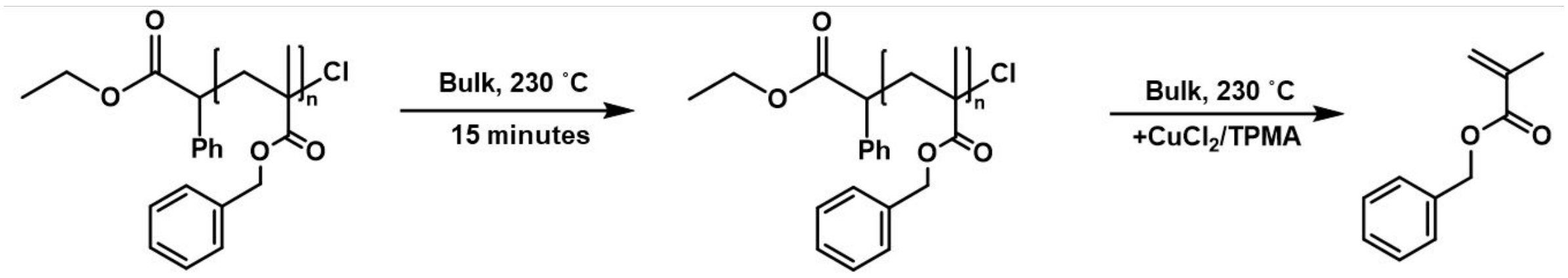

Scheme S4: Copper catalyzed bulk depolymerization of PBzMA after a 5-minute incubation of the polymer in the absence of catalyst.

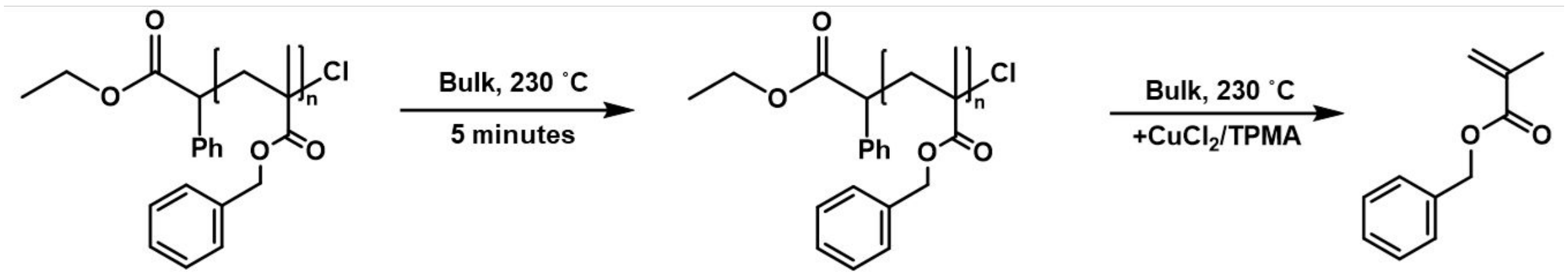

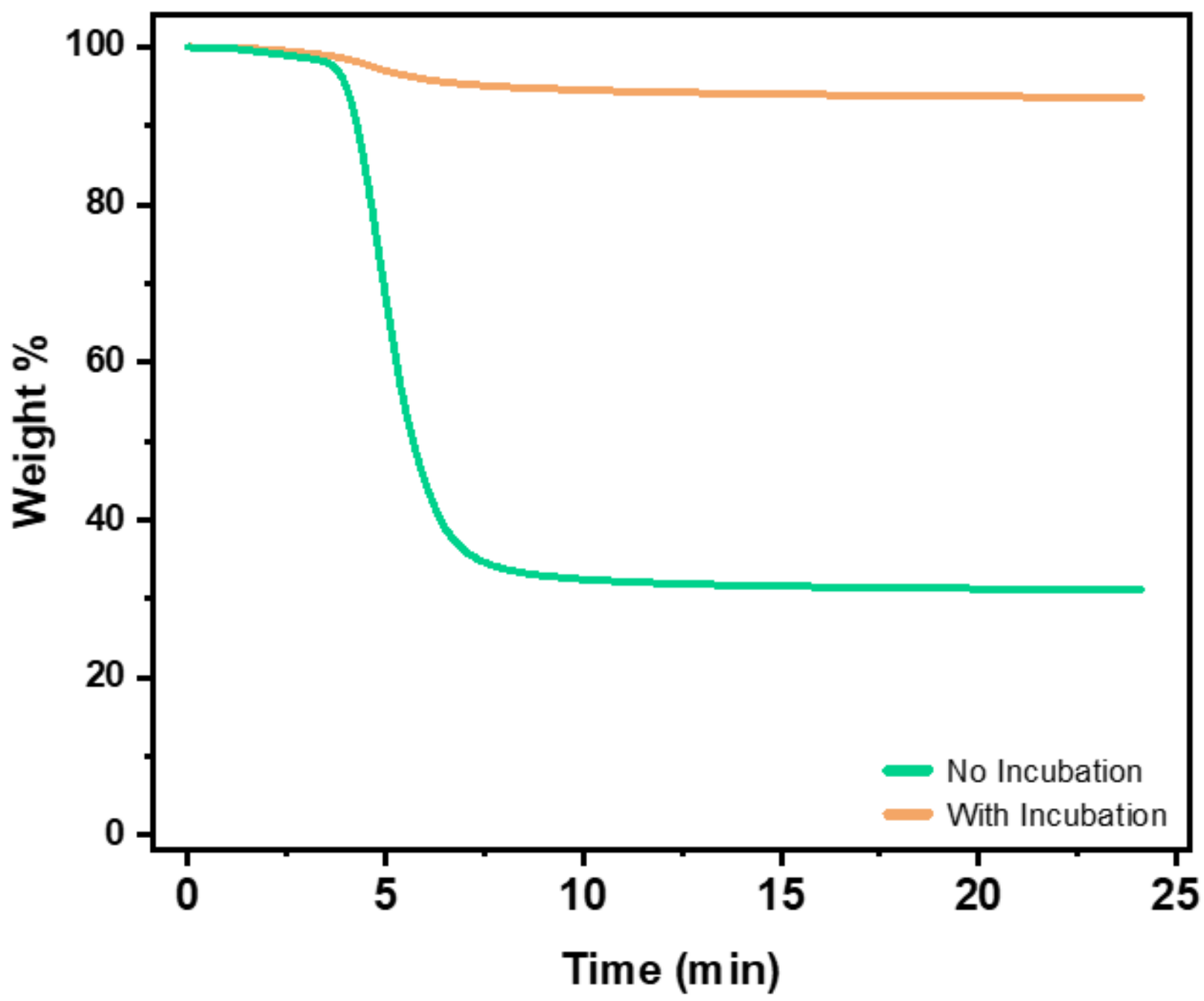

Figure S10: TGA traces showing the effect of a 5-minute incubation on the depolymerization of PBzMA. The reactions and the incubation were performed at 230˚C in the TGA instrument.

Scheme S5: Copper catalyzed bulk depolymerization of PPhMA after a 15-minute incubation of the polymer in the absence of catalyst.

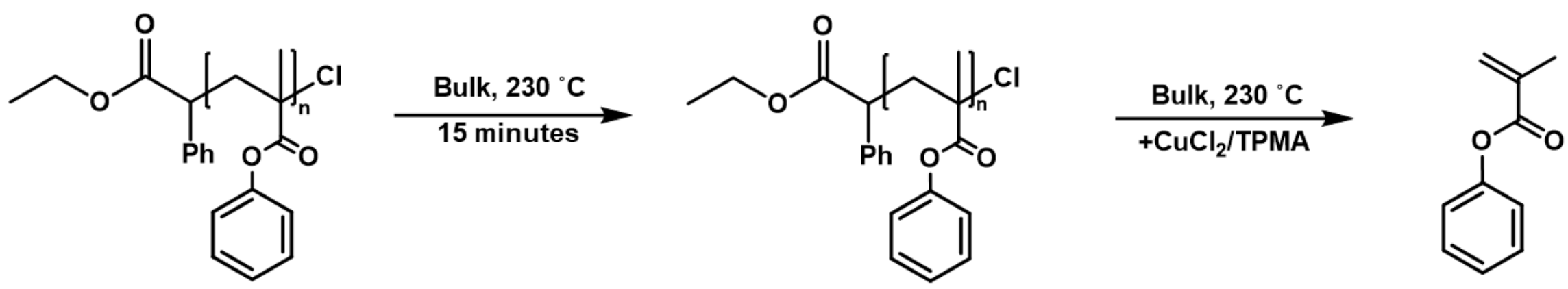

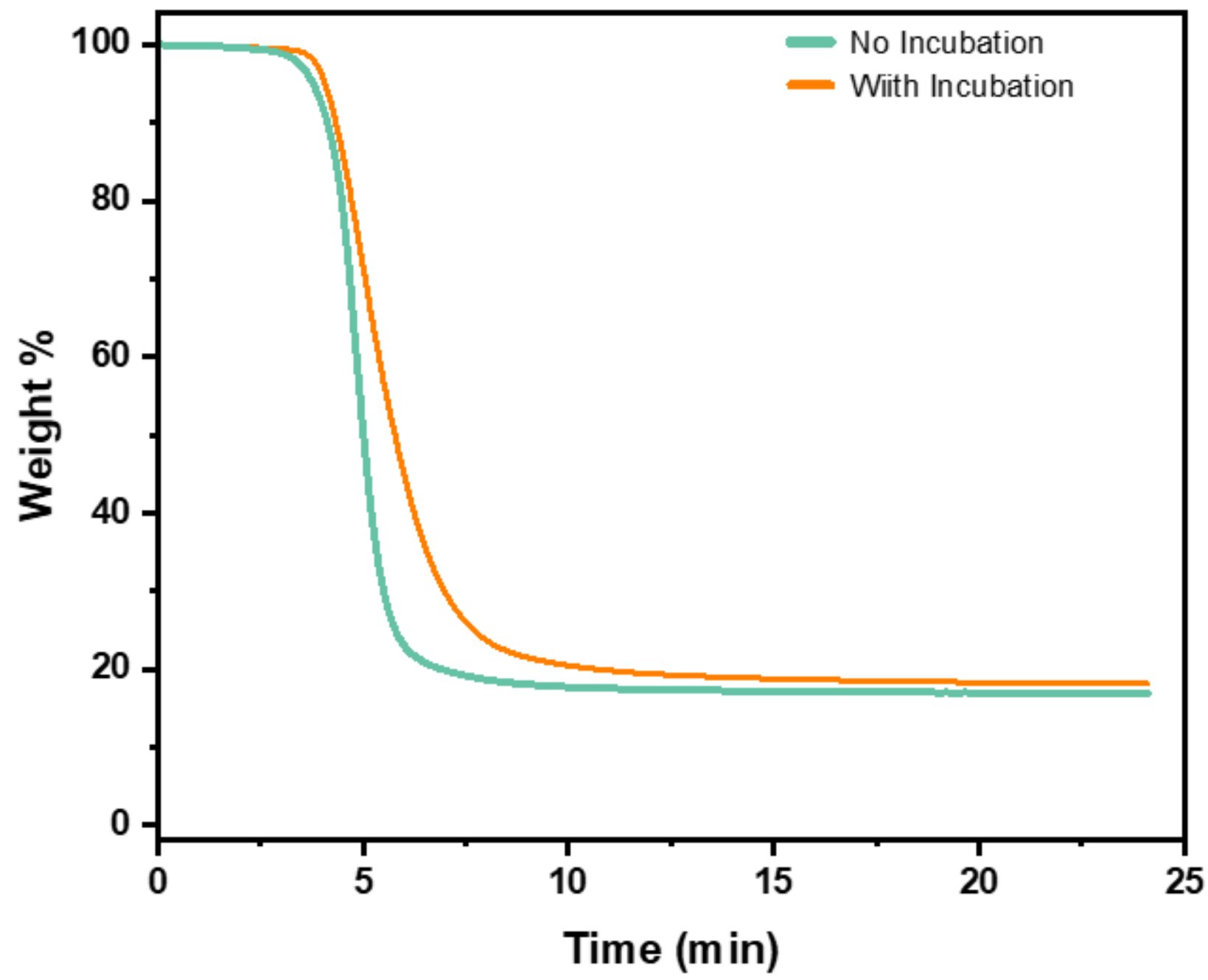

Figure S11: TGA traces showing the effect of a 15-minute incubation on the depolymerization of PPhMA. The reactions and the incubation were performed at 230˚C. The reactions and the incubation were performed at 230˚C in the TGA instrument. The depolymerization without incubation was 83% and with incubation was 82%.

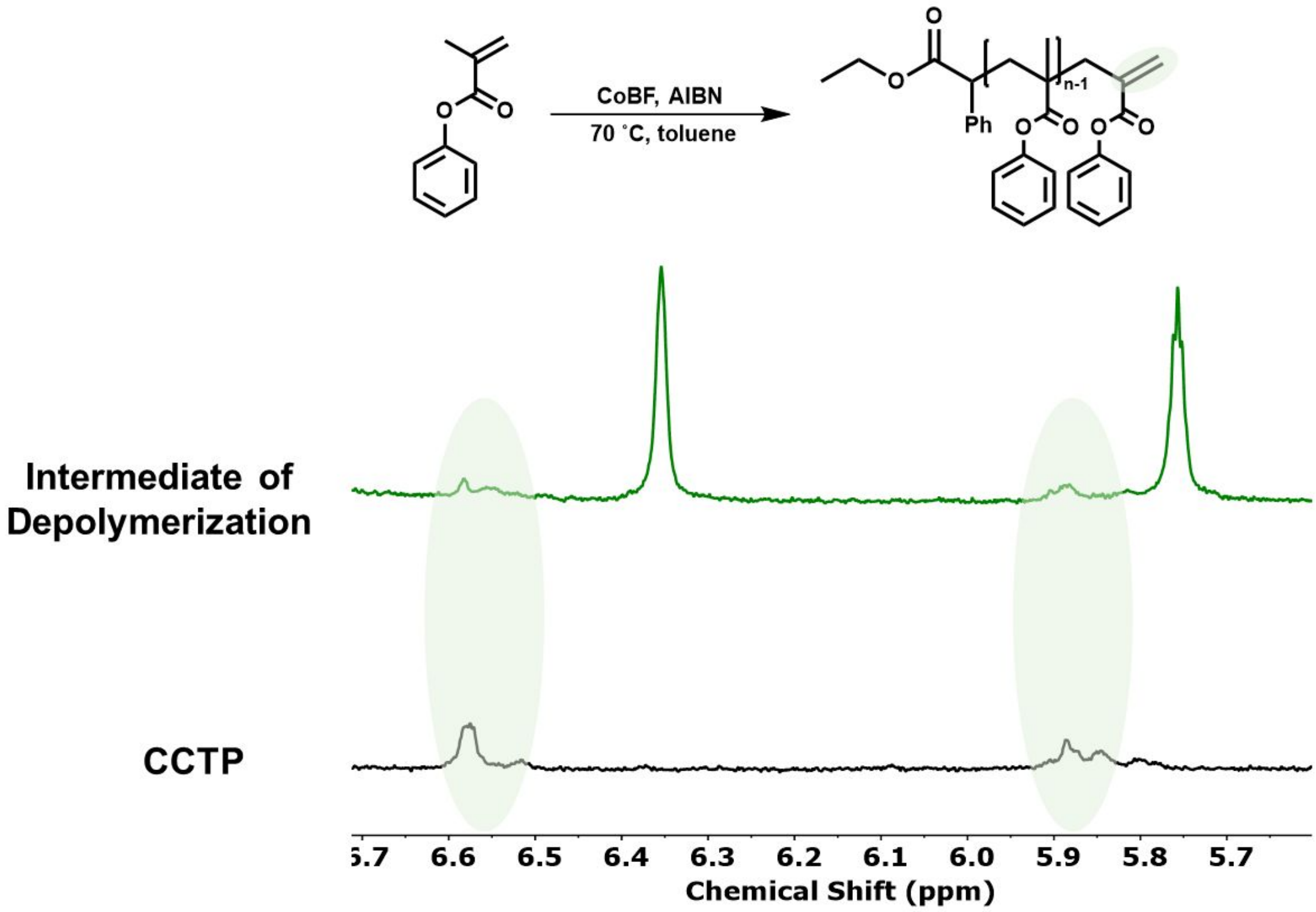

Figure S12: ^{1}H NMR data of PPhMA obtained by CCTP. Zoomed in is the region between 5.6 and 6.9 ppm and highlighted are the distinct vinyl peaks present in macromonomer (black). This is compared to the intermediate in the catalyst-free depolymerization reaction (green).

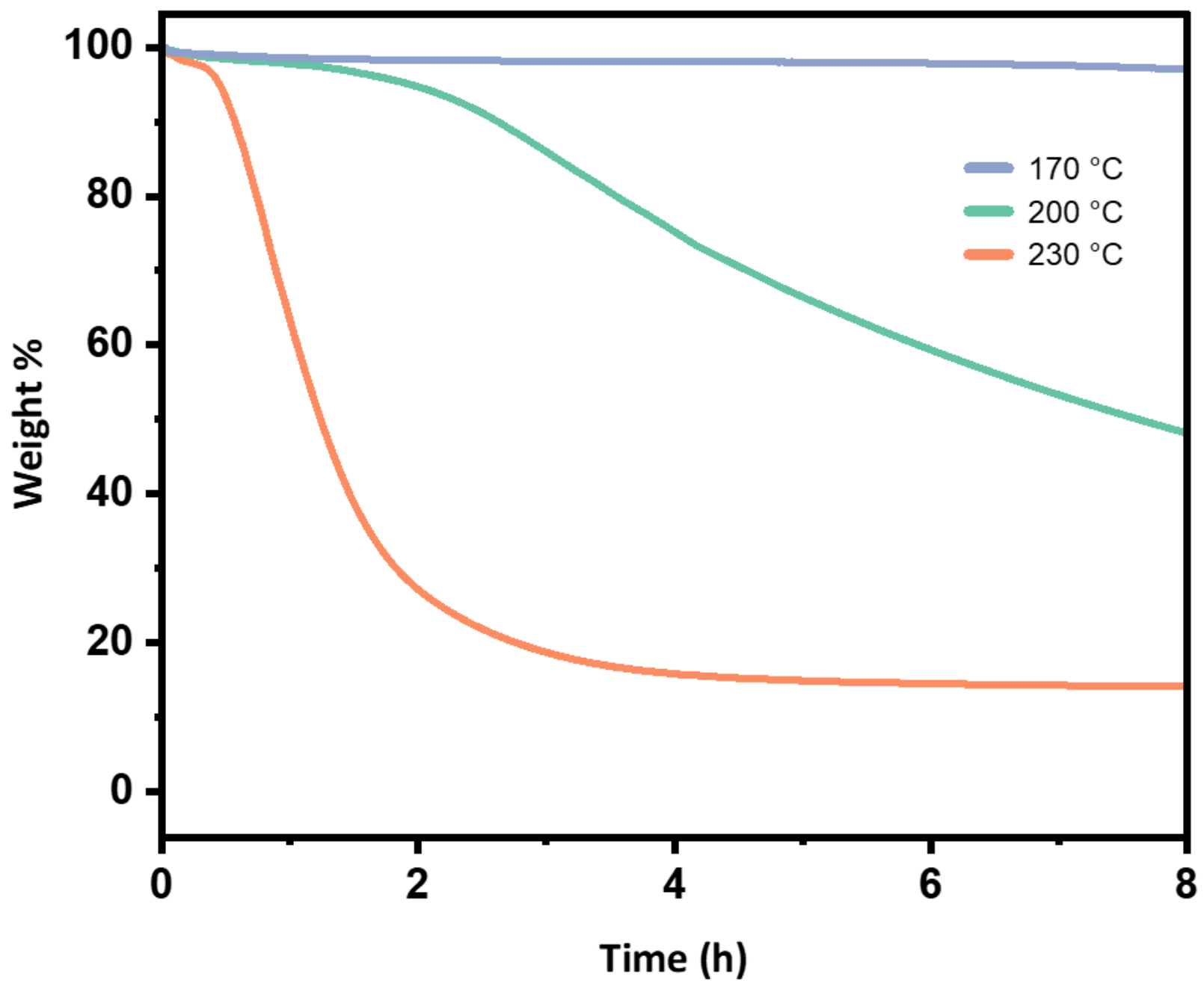

Figure S13: TGA spectra after heating PPhMA to 230˚C (orange), 200˚C (green) and 170˚C (blue), respectively. Reactions were performed for 8 hours in the absence of catalyst.

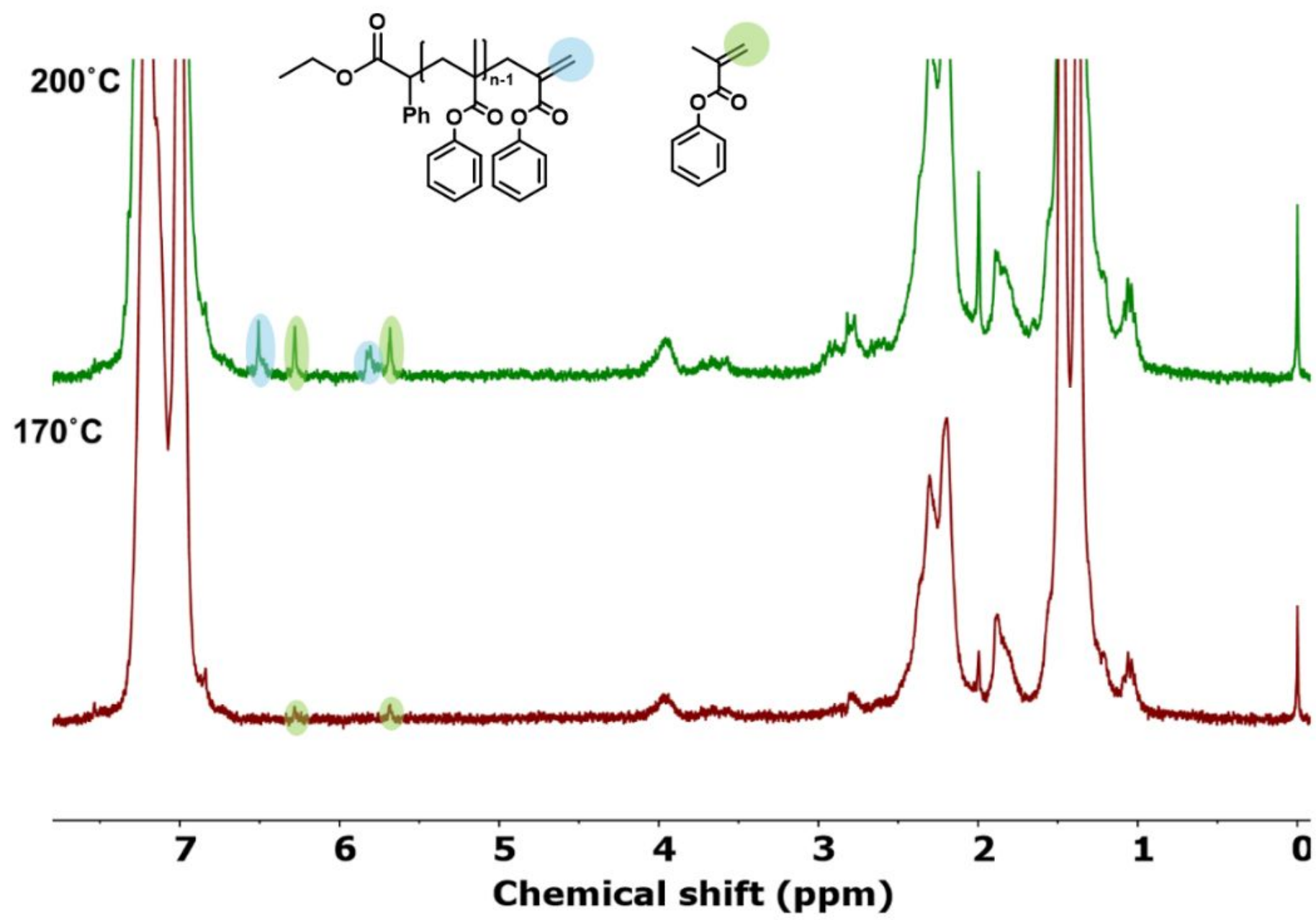

Figure S14: ^{1}H NMR spectra after heating PPhMA to 200˚C (green) and 170˚C (red), respectively. Macromonomer formation is observed at 200˚C but not 170˚C (peaks at 5.9 and 6.6 ppm).

Solution Depolymerization of PBzMA and PPhMA

Scheme S6: Attempted catalyst-free depolymerization of PBzMA in solution.

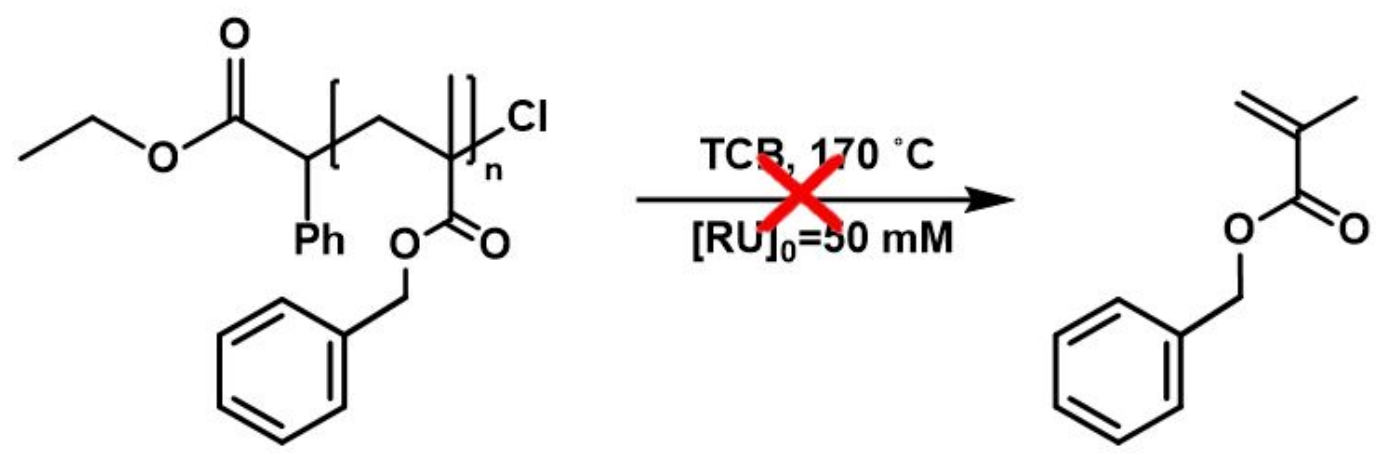

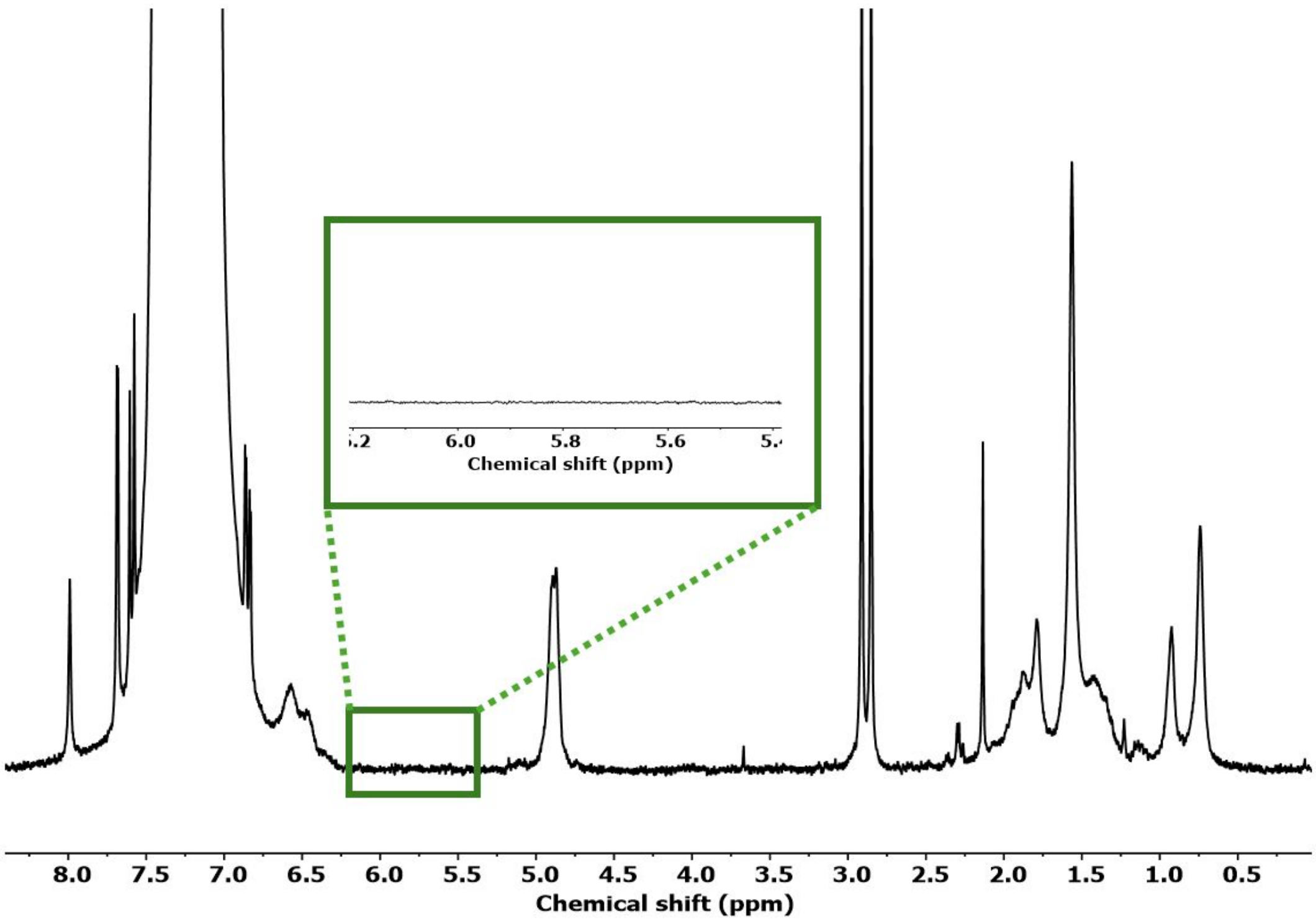

Figure S15: ^{1}H NMR showing the results of the attempted catalyst-free solution depolymerization of PBzMA. The zoomed in region between 5.5 and 6.2 shows the absence of any monomer. Reaction was performed with a $[RU]_0$ concentration of 50 mM in TCB at 170˚C.

Scheme S7: Attempted catalyst-free depolymerization of PPhMA in solution.

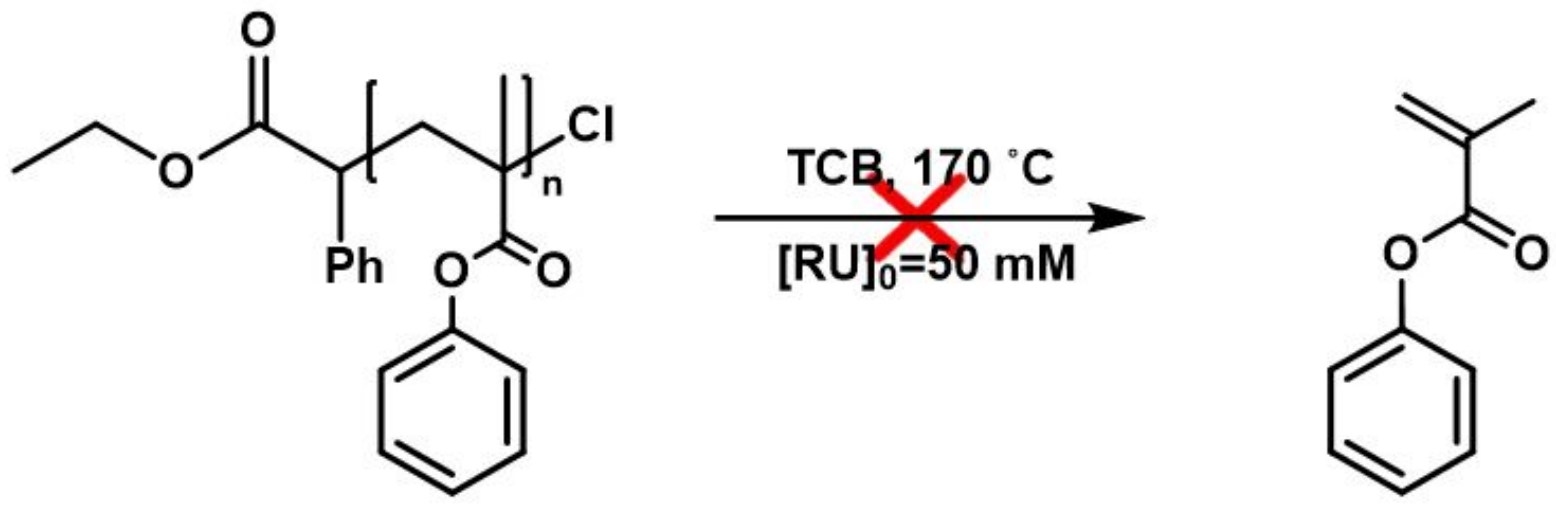

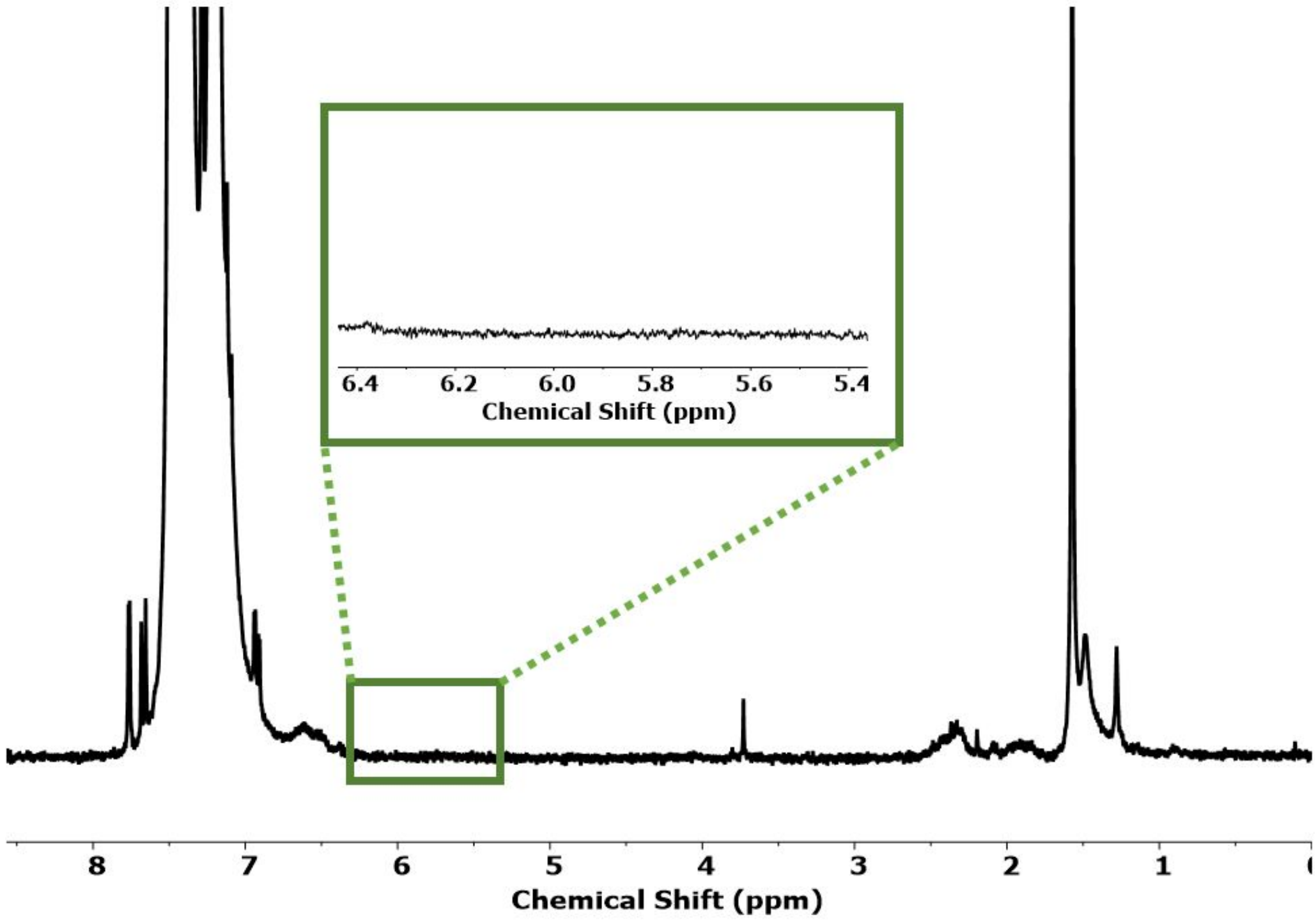

Figure S16: ^{1}H NMR showing the results of the attempted catalyst-free solution depolymerization of PBzMA. The zoomed in region between 5.4 and 6.4 shows the absence of any monomer. Reaction was performed with a $[RU]_0$ concentration of 50 mM in TCB at 170˚C.

Scheme S8: Solution depolymerization of PBzMA after a 24-hour incubation of the polymer in the absence of catalyst.

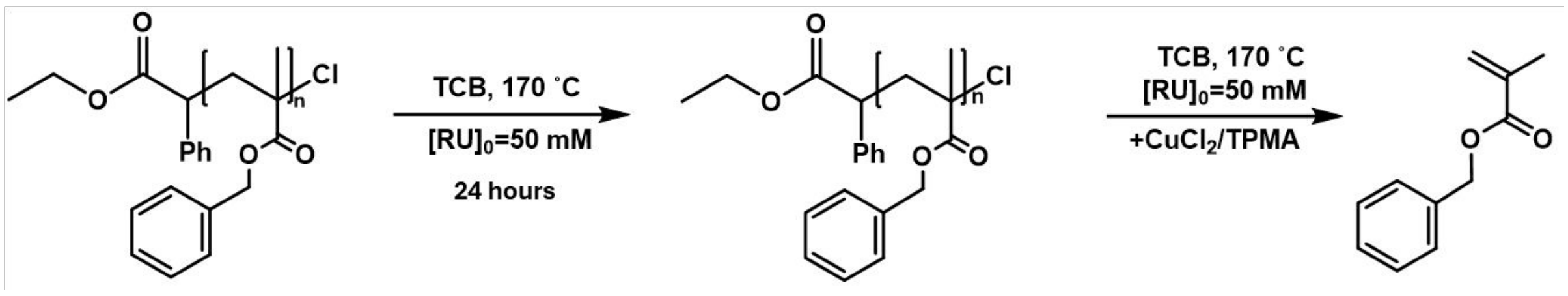

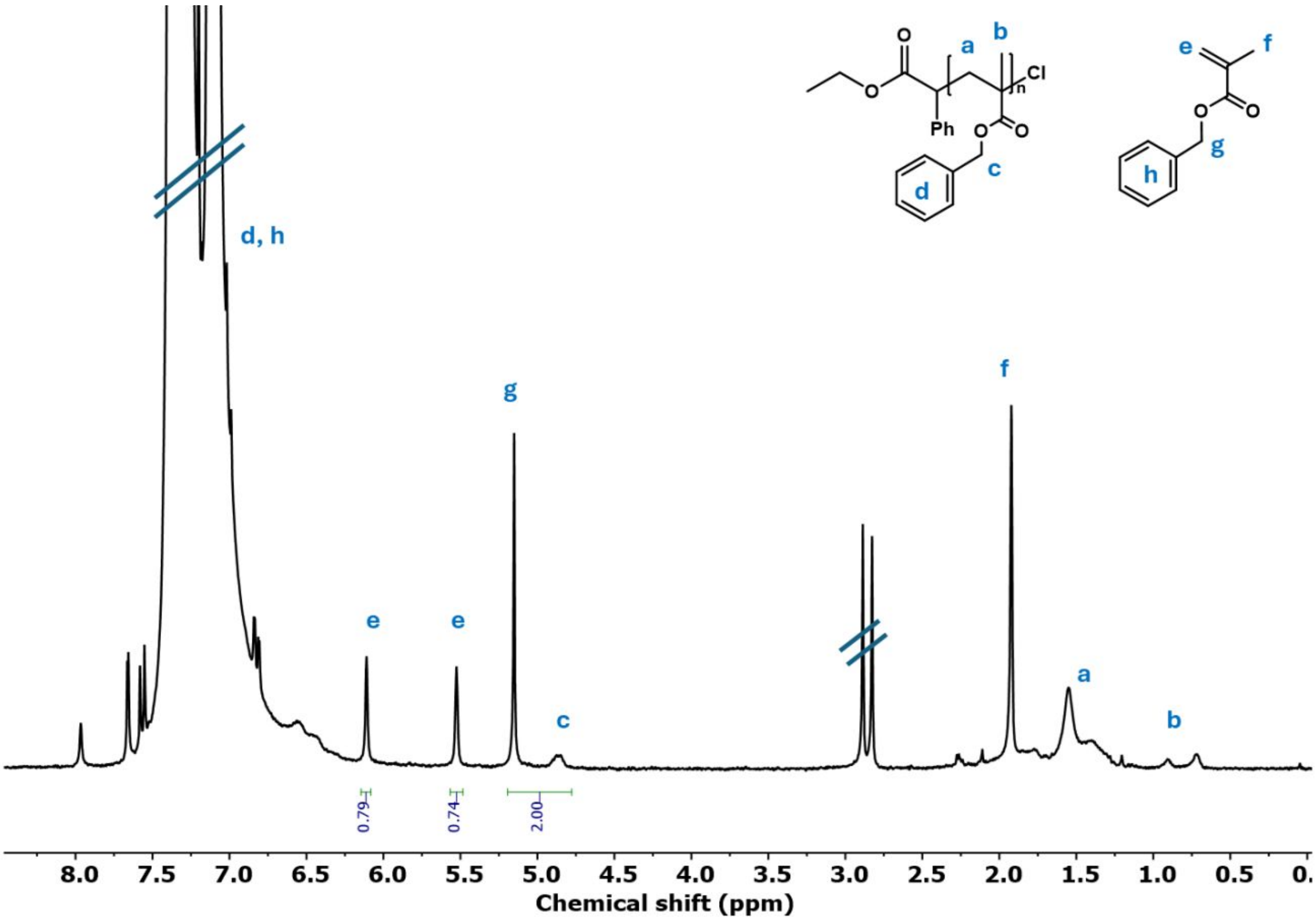

Figure S17: ^{1}H NMR showing the results of the solution depolymerization of PBzMA after a 24-hour incubation and subsequent introduction of catalyst. Reaction was performed with a $[RU]_0$ of 50 mM in TCB at 170˚C. After 24 hours, $CuCl_2$/TPMA was added giving a ratio of [PBzMA]:[$CuCl_2$]:[TPMA]=1:0.22:1.3. A sample was taken after 15 further minutes of reaction time.

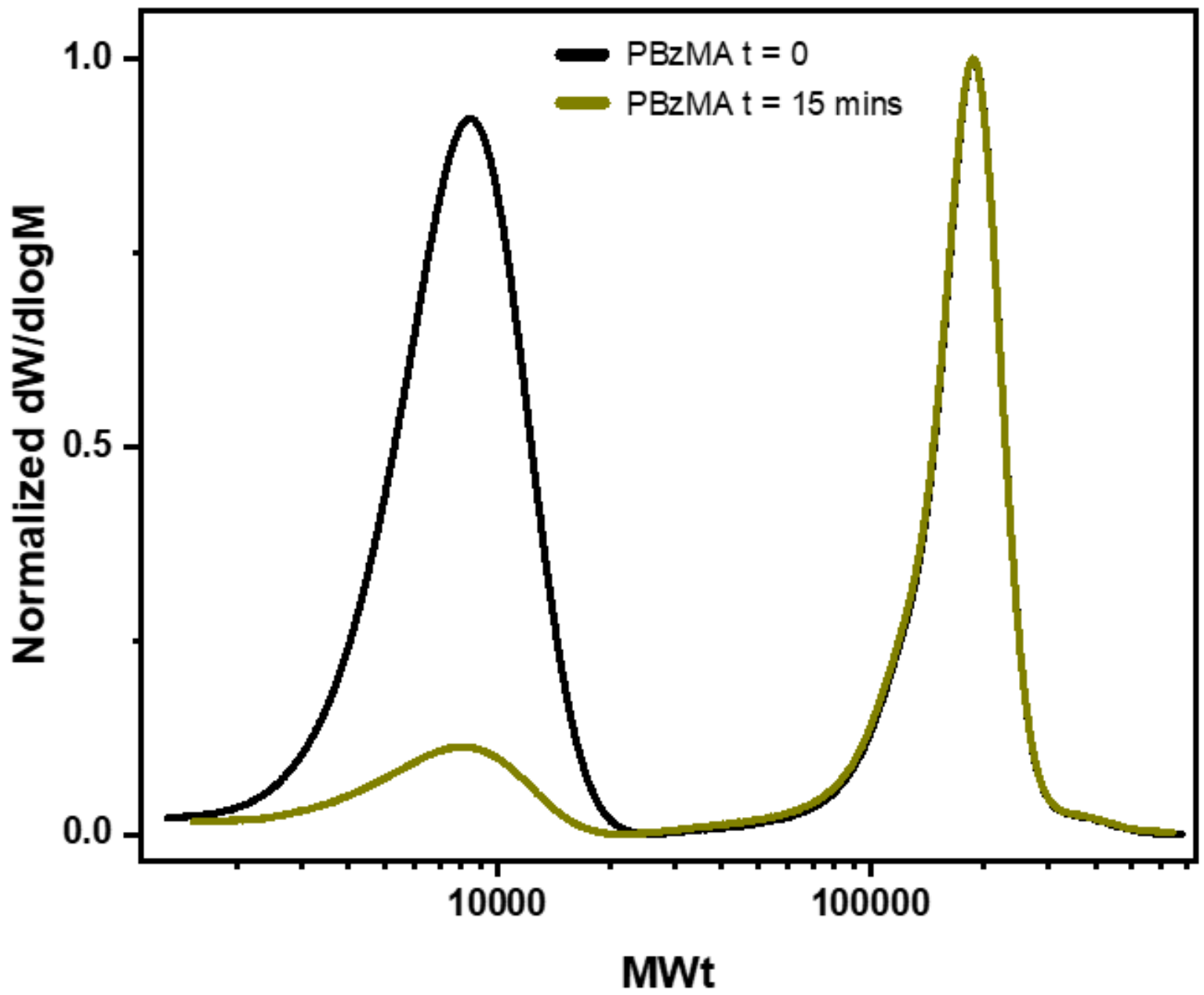

Figure S18: SEC traces for the solution depolymerization of PBzMA after a 24-hour incubation and subsequent introduction of catalyst. Reaction was performed with a $[RU]_0$ of 50 mM in TCB at 170˚C. After 24 hours, $CuCl_2$/TPMA was added in a ratio of [PBzMA]:[$CuCl_2$]:[TPMA]=1:0.22:1.3. A sample was taken after 15 further minutes of reaction time. The high molecular weight polymer was a PS standard that would not react during the depolymerization. Time zero is when the catalyst was added to the reaction.

Scheme S9: Solution depolymerization of PPhMA after a 24 hour incubation of the polymer in the absence of catalyst.

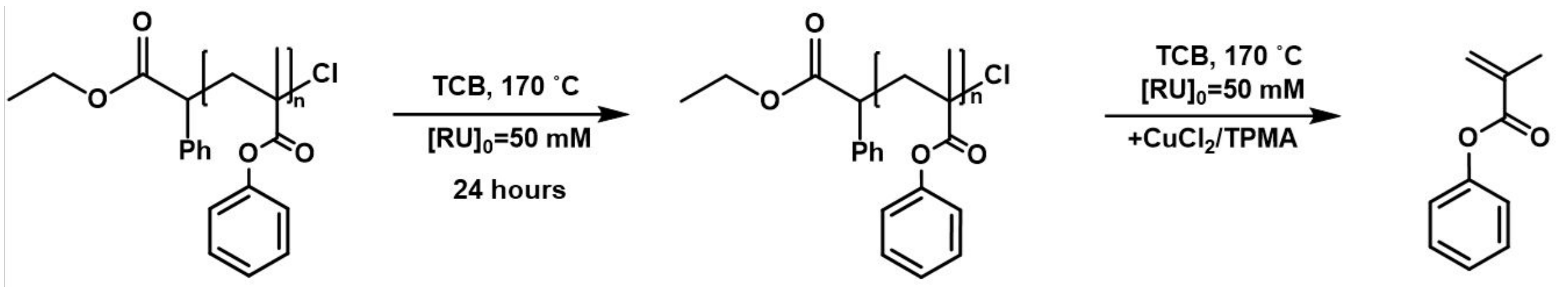

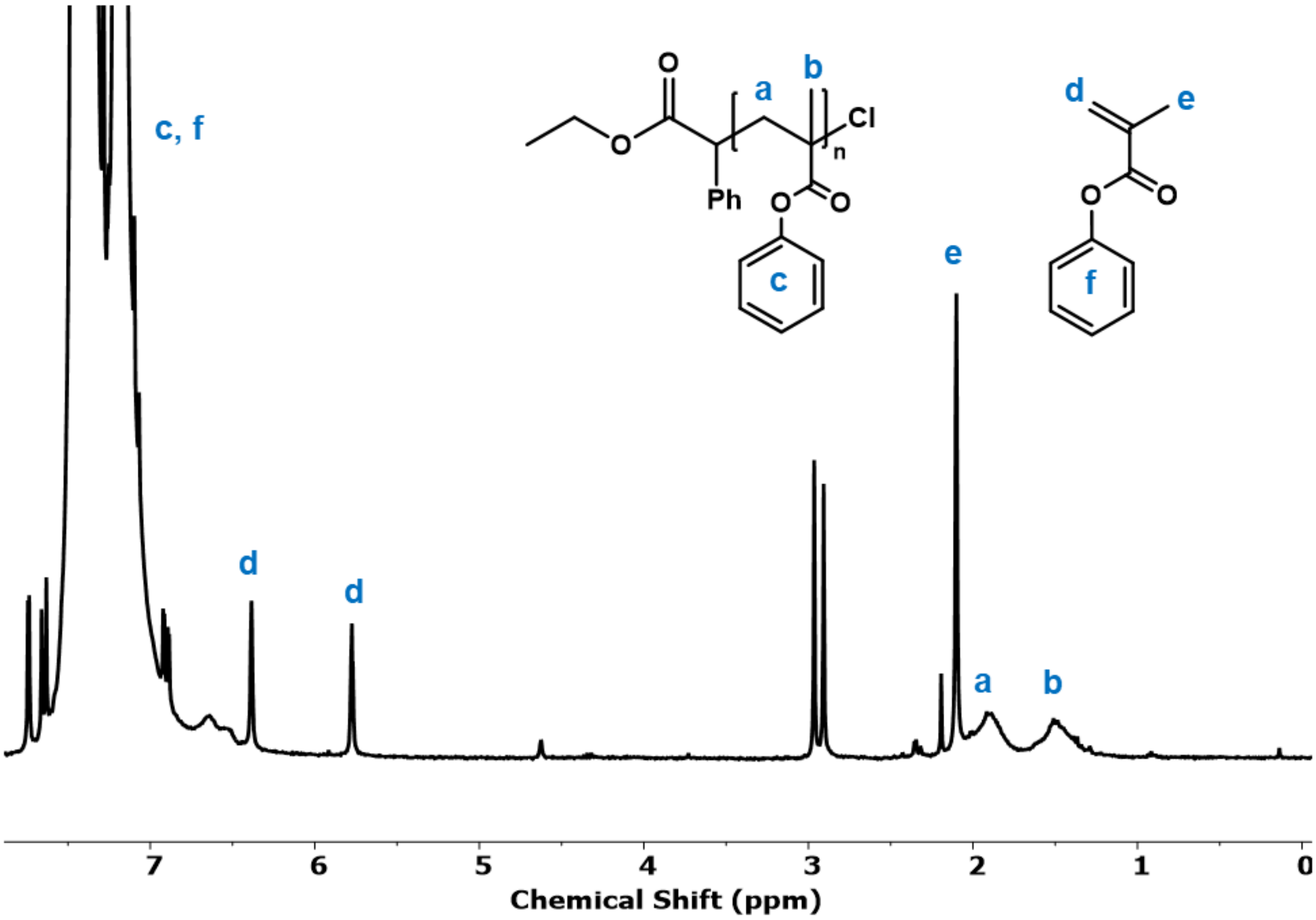

Figure S19: ^{1}H NMR showing the results of the solution depolymerization of PPhMA after a 24-hour incubation and subsequent introduction of catalyst. Reaction was performed with a $[RU]_0$ of 50 mM in TCB at 170˚C. After 24 hours, $CuCl_2$/TPMA was added in a ratio of [PPhMA]:$[CuCl_2]$:[TPMA]=1:0.22:1.3. A sample was taken after 15 further minutes of reaction time.

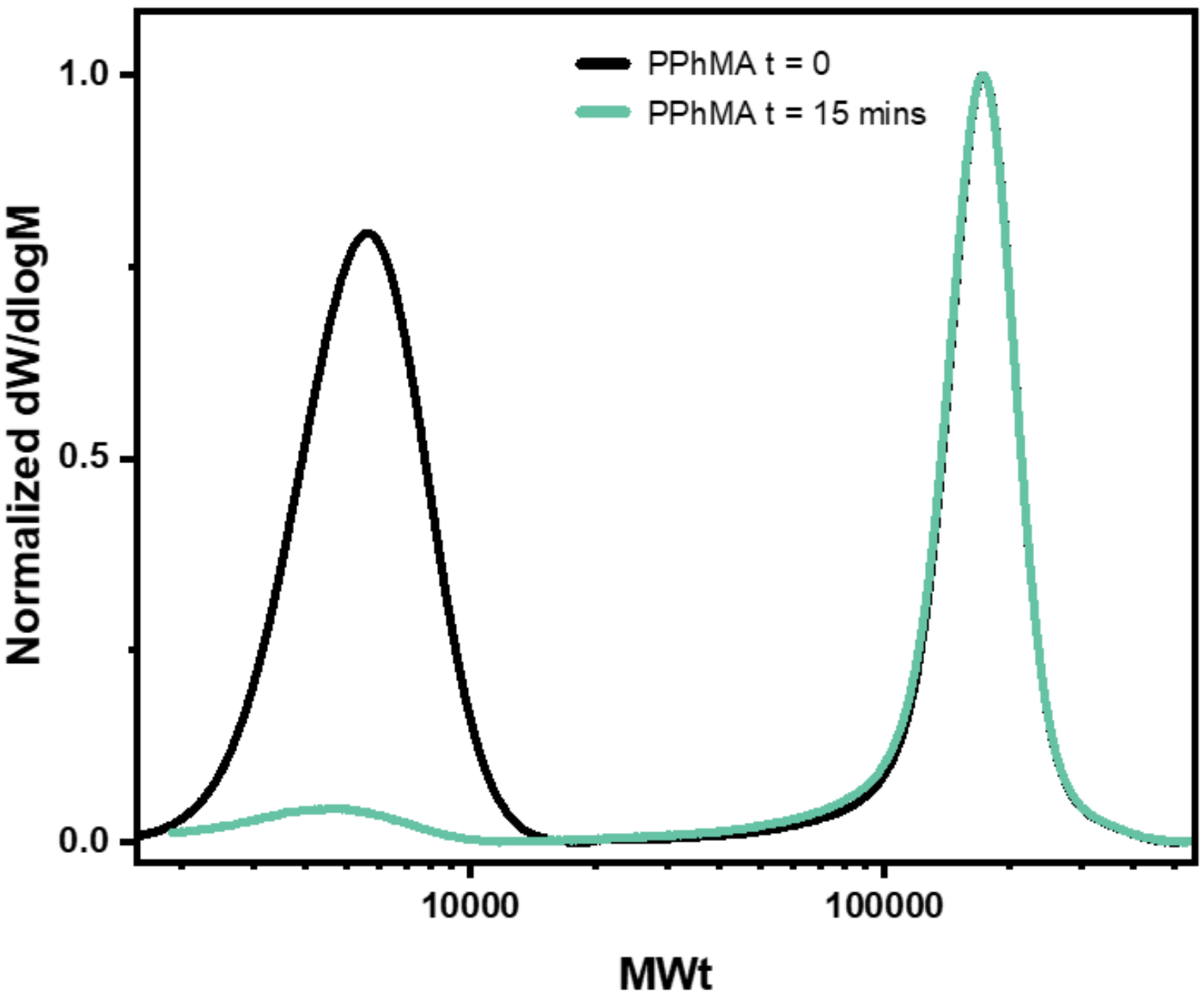

Figure S20: SEC traces for the solution depolymerization of PPhMA after a 24-hour incubation and subsequent introduction of catalyst.. Reaction was performed with a $[RU]_0$ of 50 mM in TCB at 170˚C. After 24 hours, $CuCl_2$/TPMA was added in a ratio of [PPhMA]:[$CuCl_2$]:[TPMA]=1:0.22:1.3. A sample was taken after 15 further minutes of reaction time. The high molecular weight polymer was a PS standard that would not react during the depolymerization. Time zero is when the catalyst was added to the reaction.

Catalyst-Free Bulk Depolymerization of Conventional Polymethacrylates

Scheme S10: Scheme illustrating the synthesis of P(BzMA-b-PPhMA) via ARGET ATRP. A ratio of [PBzMA]:[PhMA]:[$CuCl_2$]:[PMDETA]:[$Sn(eh)_2$] = 1:20:0.1:0.15:0.2 was used with anisole as the solvent and a temperature of 70˚C.

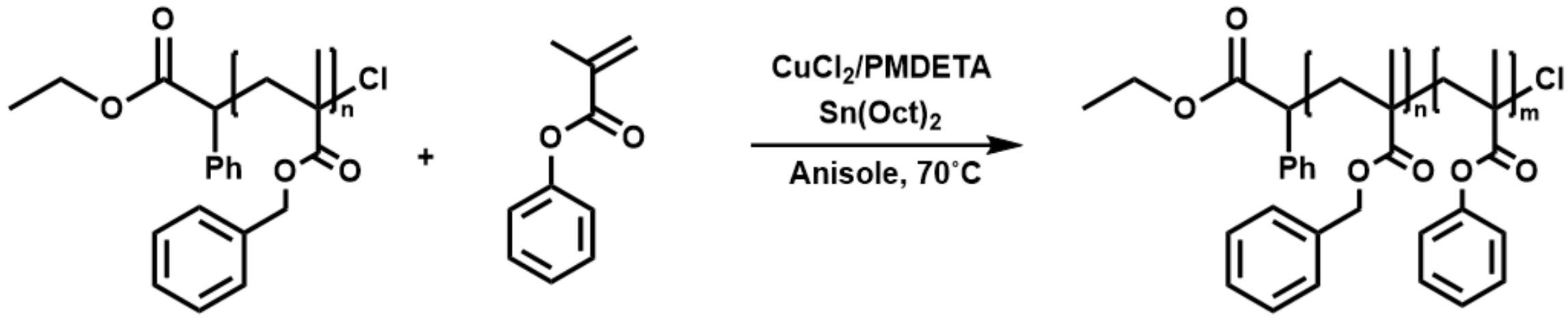

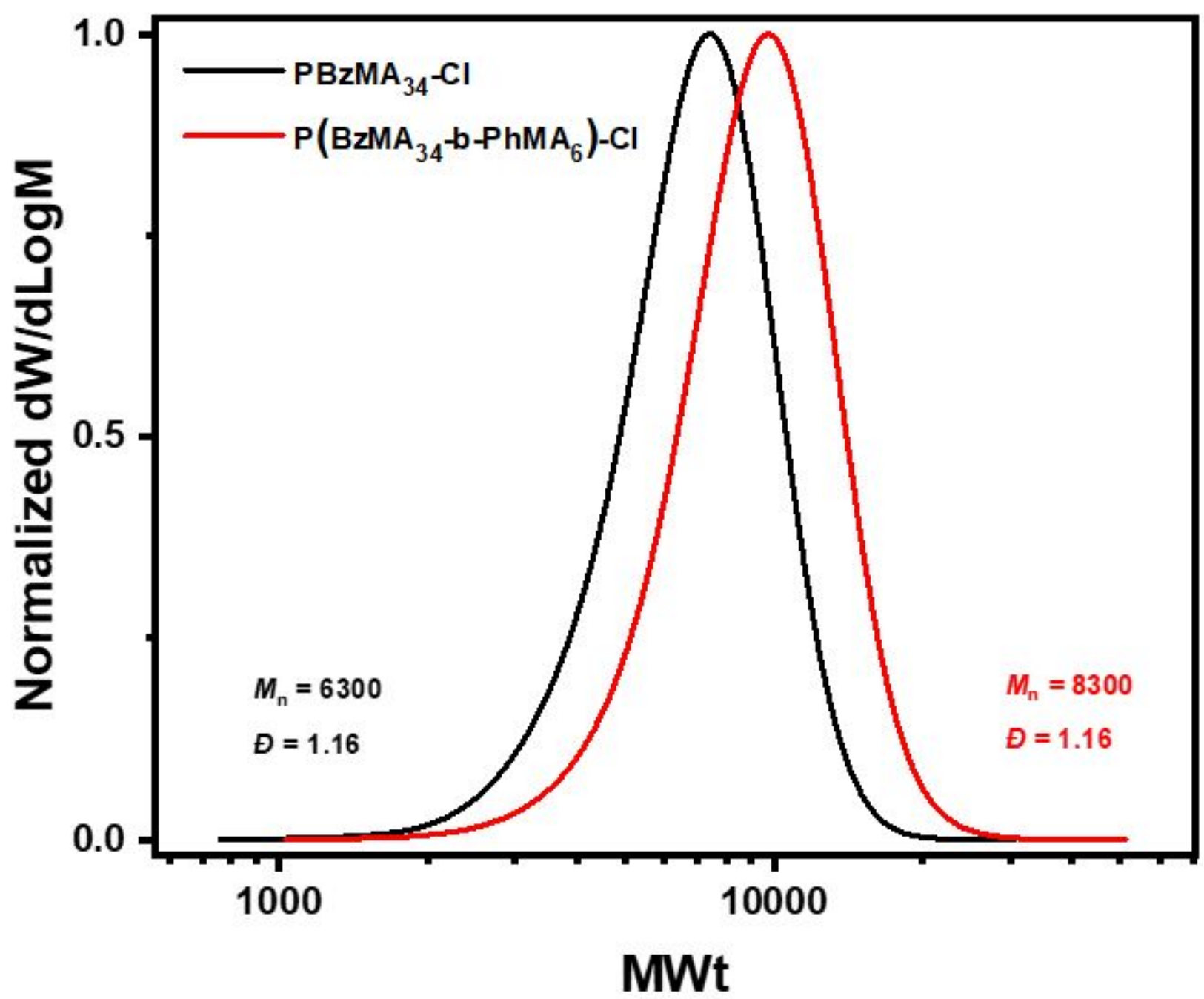

Figure S21: SEC trace of the purified P(BzMA-b-PPhMA).

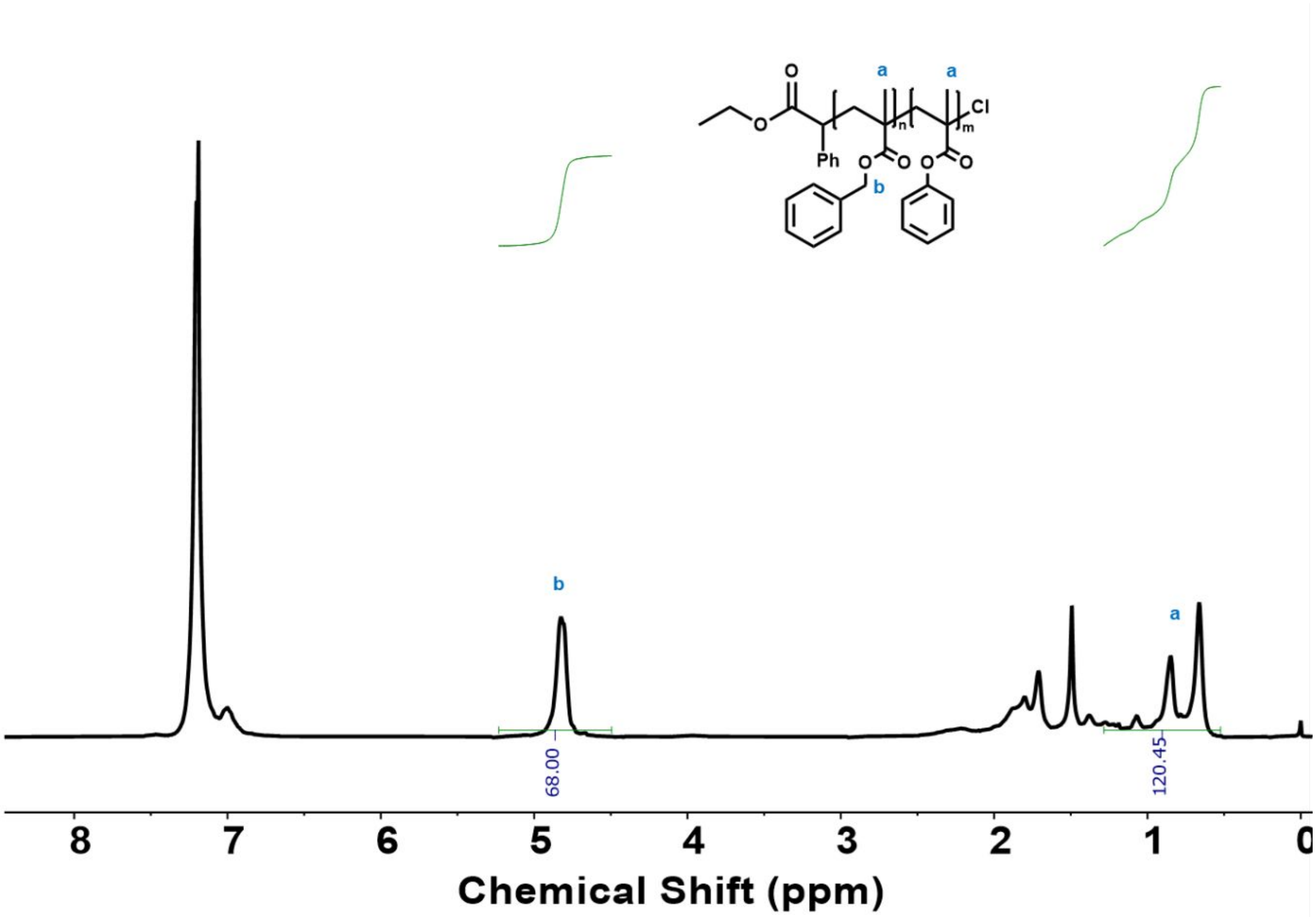

Figure S22: ^{1}H NMR of the purified P(BzMA-b-PPhMA).

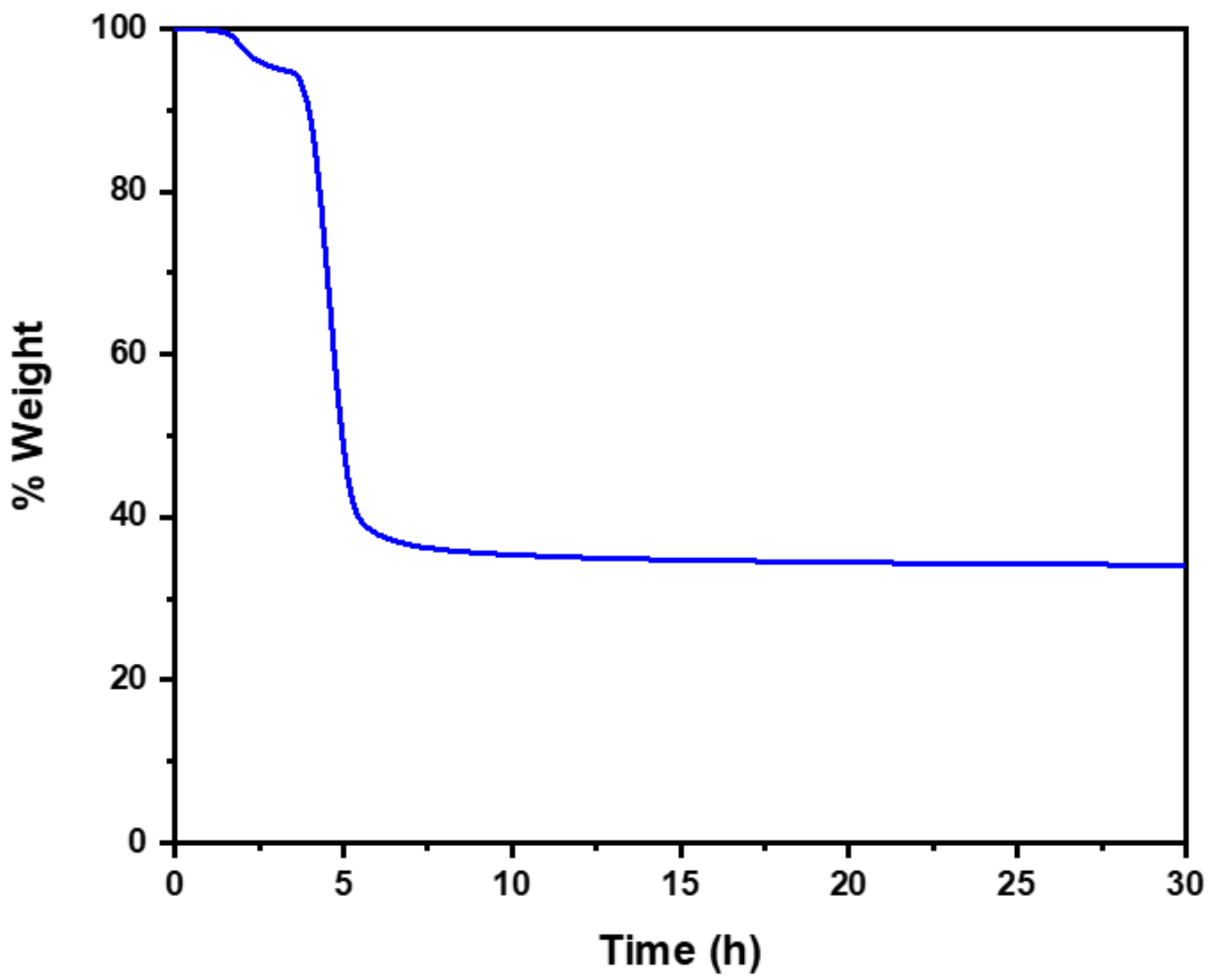

Figure S23: TGA analysis for the $CuCl_2$/TPMA catalyzed depolymerization of P(BzMA-b-PhMA). The conversion was 67% compared to 66% for the catalyst-free reaction. Reaction was performed at 230˚C with a ratio of [P(BzMA-b-PhMA)]:[$CuCl_2$]:[TPMA] = 1:0.22:0.22.

Scheme S11: Scheme illustrating the synthesis of P(MMA-b-PPhMA) via ARGET ATRP. A ratio of [PMMA]:[PhMA]:[$CuCl_2$]:[PMDETA]:[$Sn(eh)_2$] = 1:20:0.1:0.15:0.2 was used with anisole as the solvent and a temperature of 70˚C.

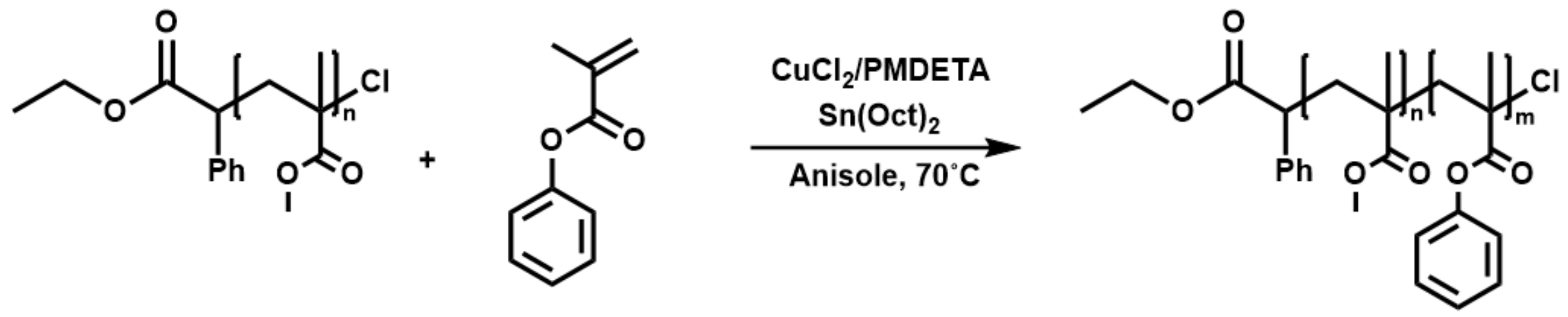

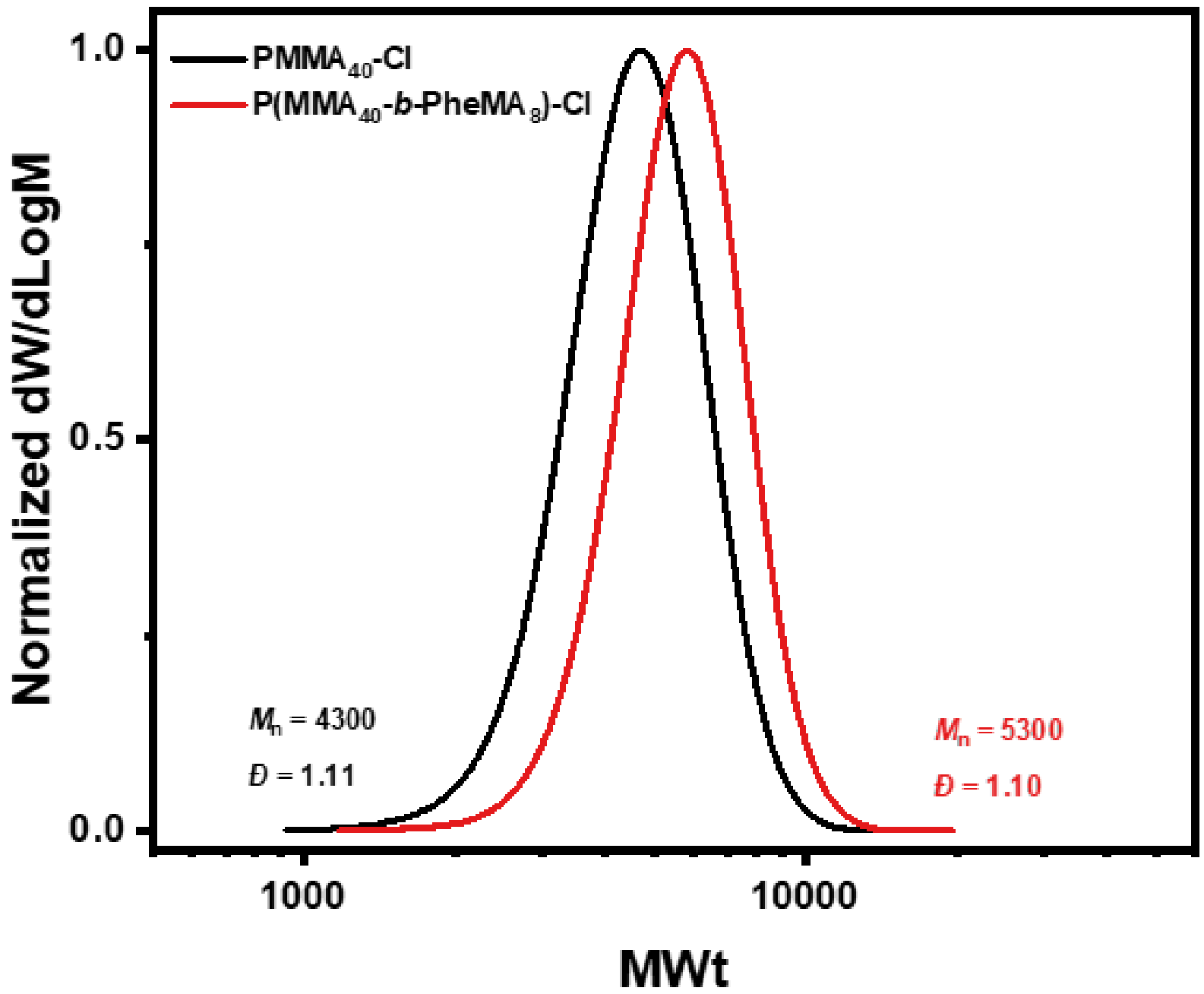

Figure S24: SEC trace of the purified P(MMA-b-PPhMA).

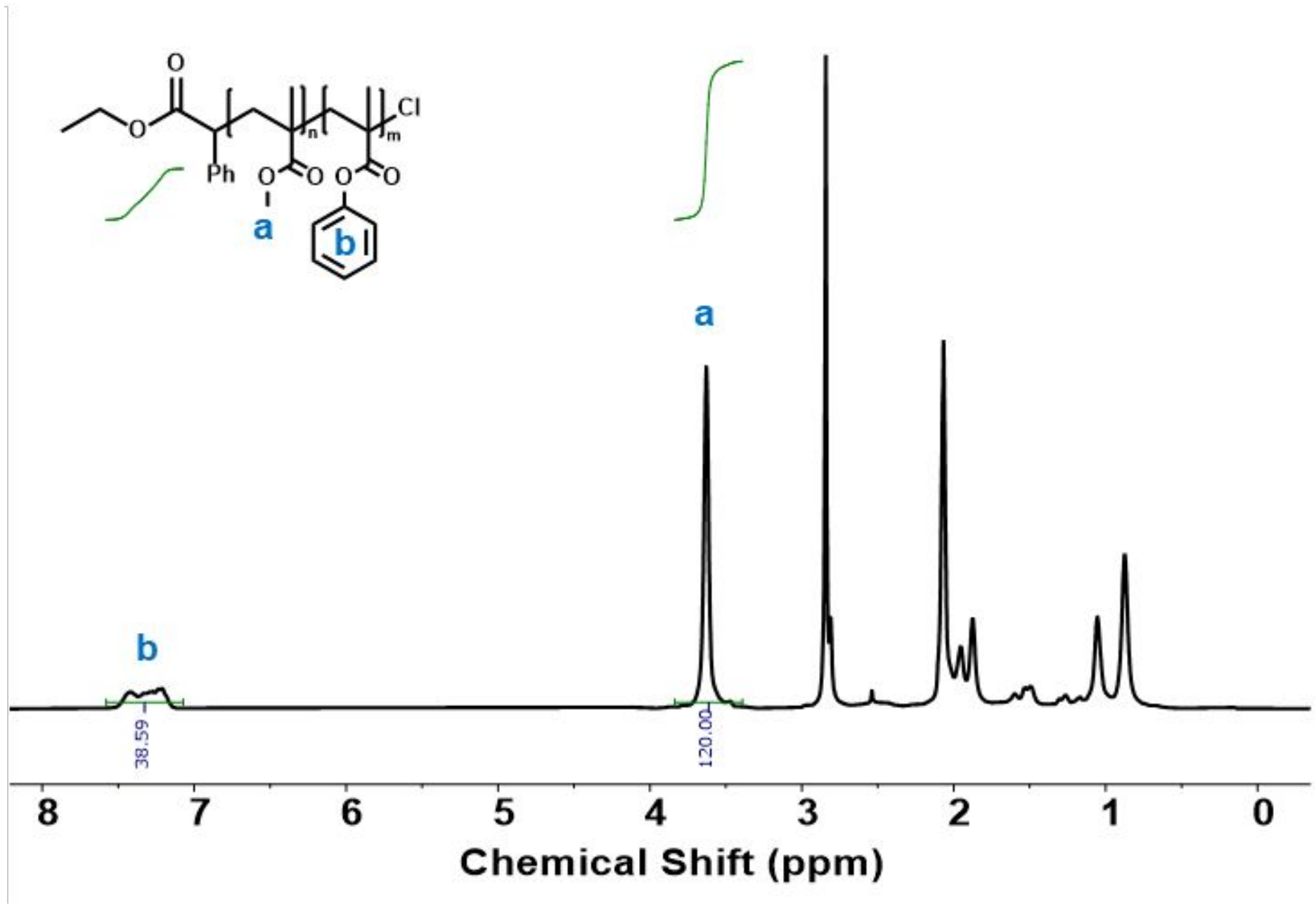

Figure S25: ^{1}H NMR of the purified P(MMA-b-PPhMA).

References

1. Bartels, J. W.; Cauët, S. I.; Billings, P. L.; Lin, L. Y.; Zhu, J.; Fidge, C.; Pochan, D. J.; Wooley, K. L., Evaluation of Isoprene Chain Extension from PEO Macromolecular Chain Transfer Agents for the Preparation of Dual, Invertible Block Copolymer Nanoassemblies. *Macromolecules* **2010,** *43*, 7128-7138.
